# Factors Influencing the Biological Effects of FLASH Irradiation

**DOI:** 10.3390/antiox14111372

**Published:** 2025-11-19

**Authors:** Sergey Igorevich Glukhov, Elena Ananievna Kuznetsova, Sergey Vsevolodovich Akulinichev

**Affiliations:** 1 Institute for Nuclear Research, Russian Academy of Sciences (INR RAS), Moscow 108840, Russia; kuzglu@rambler.ru (E.A.K.); akulinic@inr.ru (S.V.A.); 2 Institute of Theoretical and Experimental Biophysics, Russian Academy of Sciences (ITEB RAS), Pushchino 142290, Russia; 3 State Scientific Center “B.V. Petrovsky Russian Scientific Center of Surgery” (B.V. Petrovsky RSCS), Moscow 119991, Russia

**Keywords:** FLASH-effect, FLASH-RT, electrons, protons, helium ions, carbon ions, X-rays, animal models, ROS, oxidative stress, hypoxia, oxygen depletion, anesthesia

## Abstract

Among the methods for increasing the specificity of tumor radiotherapy, FLASH radiotherapy (FLASH-RT) stands out, having recently entered clinical trials. A distinctive feature of this treatment method is the delivery of a therapeutic dose in a fraction of a second with a typical mean dose rate greater than 40 Gy/s. In addition to improved patient comfort and a shorter hospital stay, this therapy potentially carries a lower risk of radiation-related side effects due to reduced damage to normal tissues. Numerous preclinical and in vivo laboratory trials of FLASH-RT have demonstrated that, in addition to reducing the severity of radiation-related complications, FLASH radiotherapy has antitumor efficacy similar to conventional radiotherapy. Partly reduced radiotoxicity after such a dose rate delivery obtained, in a broader radiobiological sense, an eponymous term FLASH effect. Although the first clinical trials aimed to evaluate the safety and efficiency of FLASH-RT against bone metastases (FAST-01/02), melanoma skin metastases (IMPulse, Flash-Skin I), Squamous Cell Carcinoma, or Basal Cell Carcinoma (LANCE) have already started or even finished and showed promising results (FAST-01), the radiobiological basis of the FLASH effect is far from a complete explanation. The fundamental factors explaining the nature of the FLASH effect are mainly considered to be the following: (1) changes in the balance of water radiolysis products and a decrease in the generation of stable reactive oxygen species (ROS), (2) differential oxygen depletion, depending on the initial oxygen concentration in tissues, and (3) physiological and metabolic, gene expression and probably epigenetic shifts in response to irradiation in normal and tumor cells. The main purpose of this review is the systematization of the radiobiological manifestations of the FLASH effect together with a consideration of the elementary processes laying in the basis of the FLASH effect in order to actualize rationale and future application developments of FLASH-RT.

## 1. Introduction

Radiation therapy has traditionally been one of the main treatment methods for primary tumors and their metastases. Unfortunately, like most cancer treatments, radiation therapy is not without its drawbacks, including incomplete response of cancer cells to therapy, radiation damage to normal tissues in the beam area, and the development of post-radiation complications such as dermatitis, fibrosis, and ulcers. No less dangerous is the significant risk of developing secondary cancers that arise after undergoing a course of radiotherapy, thus is making a search for strategies to increase the effectiveness of radiotherapy vital [1]. Among the proposed approaches to increasing the effectiveness of radiation therapy, along with methods for improving the conformality of tumor irradiation, increasing the radiation dose rate has been proposed in recent years [2]. Unlike conventional radiotherapy (CONV-RT) with an average dose rate of several Gy/min, recently suggested promising FLASH radiotherapy (FLASH-RT), proposes using an average dose rate of several tens to thousands of Gy/s. Such a dose rate with a fraction volume suitable for radiobiological in vitro and in vivo biomedical and veterinary modeling is also known as “FLASH irradiation” or “FLASH mode” in the extended radiobiological literature.

The expected advantage of FLASH-RT is reduced damage to normal tissue while maintaining toxicity to tumor cells, now considered a property of the ultra-high dose irradiation (UHDR)—(Table 1) mode now known as the FLASH effect [2]—is being actively studied for clinical use.

According to the findings of a significant number of studies, dose delivery in FLASH mode results in reduced radiation damage or mortality in laboratory animals (Table 2).

Irradiation of animal models in FLASH mode led to less damage to actively dividing cells in tissues that traditionally have a higher radiation sensitivity, and, as a consequence, to a lesser development of post-radiation fibrosis [26,57,68], general inflammation [37,69,70], and pyroptosis [57]. Interestingly, in vivo experiments showed that the FLASH mode resulted in less damage to normal cells in healthy organs while causing similar damage to cancer cells in tumor grafts [8,13,19,20,23,30], while in vitro experiments generally showed greater survival of both tumor and normal cell lines after FLASH irradiation [71,72,73,74]. In some cases, the anticancer superiority of the FLASH mode over the CONV mode is also observed in experiments both in vitro [75,76] and in vivo [8,17,77,78] while simultaneously reducing radiation damage to normal tissues of tumor-bearing animals. In a number of studies, on the contrary, no FLASH effect was detected [9,18,53,56]. Furthermore, incomplete description of the physics and dosimetry may have contributed to the lack of reproducibility of radiobiological data in this field. The development of common terminology, as well as common standards for recording, reporting, dosimetry, and metrology, are currently required [79]. For the safe implementation of FLASH-RT in clinical practice, it is necessary to accurately determine and control the dose specified in the treatment plan, which is impossible to do after the fact. At the same time, technical problems associated with the development and use of adequate dosimeters and detectors must be resolved [80,81,82]. Other difficulties include the rarity and low availability of equipment for irradiation in the FLASH mode and the high cost of FLASH-RT sessions, as well as high requirements for the qualifications of specialized technical personnel who prepare the patient and irradiate a given area of the body [83,84]. Despite the identified difficulties, there is growing activity among experts and researchers in conducting preclinical studies, jointly discussing the results to solve problems for the clinical application of FLASH-RT [85,86,87,88].

The data obtained by various researchers on the effects of IR exposure to different dose rates on living objects, namely the presence of less IR-induced death of normal cells (especially actively dividing ones) after FLASH irradiation in contrast to the preservation of active death of tumor cells, also characterized by active proliferation, require careful study at all levels (physicochemical, biochemical, cellular, and organismal). According to the results of both theoretical modeling [89] and direct measurements [90,91], water radiolysis occurs in a time of about 10^−^^13^ s, which is significantly shorter than not only the time of dose delivery even in FLASH mode (fractions of a second), but also the duration of subsequent biochemical reactions of the order of μs. Therefore, the causes of the FLASH effect may be largely based on with the influence of quasi-steady-state concentrations of biologically significant radiolysis products: the level of reactive oxygen species (ROS) and activation of the associated signaling system [92], the level of hydrogen ions and local acidification of the cellular environment [93], and radiochemical depletion of oxygen is a well-known radiosensitizer. Well-oxygenated tumor areas respond to therapy three times faster than anoxic ones [94].

Due to the high interest in oxygen depletion during FLASH-mode irradiation, the level of oxygen was measured with a variable number of sensors—TROXSP5^®^ [95], OxyLite^®^ [96], raw of PtG4 [97,98,99], PdG4 [100,101,102], and 2P Oxyphor [10] sensors and the newly developed protoporphyrin IX (PpIX) [45,100,103]—primarily fluorescent with adequate FLASH-mode response time. The measurements were made as in still water [95], isotonic solution [98], or in an intracellular model—BSA solution [10,96], as in live cells [99], healthy [10,45,100,101,103], or tumor-bearing mice [10,103]

According to a number of recent experiments, it was found that the concentration of oxygen dissolved in the body’s aqueous environment plays a fundamental role in the occurrence or disappearance of the radioprotective FLASH effect [104,105]. The different oxygen concentrations in tumor and normal tissues are currently one of the main working hypotheses explaining the nature of the FLASH effect. In addition, this effect is associated with the probable participation of Fenton chemistry [106], an extra pro-glycolysis metabolism switch in the tumor with a potential to be used together with anti-glycolysis chemoterapeutical drugs [22] and the involvement of immunity when considering the FLASH effect at the organismal level [57]. However, some causes different from those mentioned above may also make sense. As the rationalization of the FLASH effect is far from completion at the present moment, as well as due to the mainly stochastic nature of reactions between variable ROS and macromolecules of cells and tissues, further studies are needed.

Equally important is the assessment of the role of the type and properties of ionizing radiation in the occurrence of the FLASH effect. Over a relatively short period of time, studies have been conducted on the biological effects of FLASH under the influence of the IR of various X-ray photons and variable particles with different linear energy transfer (LET): X-ray photons [19,30,57], electrons [67,68], protons in the plateau region [64] and the Bragg peak [58,69], helium ions [62,107], and carbon ions [74,108]. As a result, it has been shown that the general properties of lower toxicity to normal cells and tissues after irradiation in FLASH mode are present for all the studied types of IR. This can serve as confirmation of an important postulate of radiobiology about the primary importance of water radiolysis products reacting with the biochemical compounds of living organisms. Since differences in radiolysis between the pure water and cellular environment models were significant [109], the effect of FLASH irradiation on radiation-induced cell death was studied by modeling oxygen diffusion, metabolism, and radiolytic oxygen depletion in models with simulated capillary architecture. The results obtained suggest that the level of FLASH-induced radioresistance may be determined not only by the type of radiation and dose rate, but also by a combination of various physiological parameters, i.e., the FLASH effect may be tissue-specific [109,110,111,112,113].

Due to the fact that some progress has already been achieved in understanding the patterns of the FLASH effect in tumor radiotherapy, the first practical applications of FLASH-RT have taken place in veterinary medicine [32,33,34] and medicine. In 2019, the first patient with radioresistant cutaneous lymphoma was successfully treated with accelerated electrons in FLASH mode [31]. The first group of clinical trials for reducing the growth of bone metastases “FAST-01” [114] have been conducted; groups are being recruited to test the effectiveness of FLASH-RT against melanoma—“IMPulse”, “Flash-Skin I”, Squamous Cell Carcinoma or Basal Cell Carcinoma (LANCE), and bone metastases in the chest—“FAST-02” [115].

The purpose of this review is to analyze the results of several recent studies attempting to establish the physicochemical, biophysical, molecular, and physiological basis and causes of variability in the FLASH effect depending on irradiation conditions. Specifically, data are presented below on the dependence of the yield of various water radiolysis products on the type of irradiation and dose rate, the influence of cellular/tissue oxygenation, and other factors capable of altering biological effects upon exposure to IR at different dose rates.

## 2. Physicochemical Basis of the FLASH Effect

### 2.1. Radiolysis of Water Under the Influence of IR with Different Dose Rates in Model Systems

Numerous experimental data confirm the point of view that the main damaging factors when ionizing radiation impacts living organisms are the products of water radiolysis, primarily reactive oxygen species. Several hypotheses have been put forward to explain the nature of the FLASH effect, based on the characteristics of water radiolysis at ultra-high dose rates. Indeed, recent experiments in such modes have already demonstrated, at the biochemical level, less damage to DNA [116], peptides [117], and low-molecular compounds in aqueous solutions [118].

The main difficulty in registering radiolysis products is the extremely short duration of their formation and, to some extent, their lifetime. For example, the radiolysis of water by accelerated 300 MeV protons occurs in 10^−13^ s, while within 1 μs, the primary radiochemical reactions predominantly reach a quasi-steady state [89]. Various mathematical models have been proposed to describe the radiolytic depletion of oxygen under irradiation with different numbers of pulses, as well as the development of simulation models for FLASH-RT [109,119,120,121,122,123,124,125,126,127]. Monte Carlo studies of the mechanisms of the FLASH effect have mainly tested the oxygen depletion hypotheses and intertrack radical interactions [97,106,109,111,126,127,128,129]. Intertrack radical reactions are more accurate for UHDR mode where the concentration of radicals in a moment is too high to be able to diffuse from one track of origin to another, whereas low-dose delivery rate radical reactions are the overwhelming majority and commonly take place in an intra-track space separated in the time tracks of non-intensive IR. Calculations show that ultra-high dose rates, due to the spatial and temporal overlapping of radical formations and mutual reactions between them, change the ratio of the number of radicals (such as, for example, hydrated electrons, hydroxyl radicals, hydrogen radicals, and hydrogen peroxide) towards a decrease in the yield of OH^•^ radicals, e_aq_^−^, H_3_O^+^, and H_2_O_2_ compared to conventional dose rates [129,130]. The FLASH effect can be influenced by the beam pulse structure and dose delivery time [110]. Direct measurement of hydrogen peroxide accumulation in water after irradiation in FLASH mode also showed its lower level [70]. The observed decrease in the H_2_O_2_ yield at high dose rates is associated, on the one hand, with a decrease in the production of OH^•^ radicals, and on the other hand, with two processes that differ in time: (1) faster (on the μs scale)—with reactions between locally concentrated radicals (e_aq_^−^, OH^•^, and H^•^) in spurs (microregions arising from the inhomogeneous distribution of radicals), which leads to the formation of less ROS; (2) a slower one (on the ms scale), which involves radiolytic molecules (H_2_O_2_ and H_2_), remaining radicals, and oxygen present in biological tissues [131,132,133]. Since different IR sources with different characteristics were used in different experiments, the results may depend on the beam structure, the number of pulses, and the duration of the time interval during which exposure and measurements were carried out [133]. When assessing the contribution of particles with different LET in experiments on direct measurements of the level of radiolysis products (H_2_, H_2_O_2_, O_2_, e_aq_^−^, H^•^, HO_2_^−^, O_2_, and OH^•^) in irradiated water equilibrated with air and contained in a closed container (Table 3), it was shown that with an increase in the LET of protons and carbon and helium ions, the yield of OH^•^ and e_aq_^−^ decreased and the yield of stable radiolysis products H_2_O_2_ and H_2_ increased [118,134,135,136,137], which cannot but contribute to the magnitude and nature of the FLASH effect.

### 2.2. Tissue Oxygenation and ROS Formation During Cell/Tissue Irradiation with Different Dose Rates

Dissolved oxygen in water is a known radiosensitizer. The second hypothesis explaining the FLASH effect is based on the phenomenon of radiation-induced oxygen depletion. It is important to note that the hydrogen peroxide accumulation values reported in Ref. [70] were obtained by irradiating water saturated with up to 4% oxygen (physioxia). Further studies have established the dynamic range of oxygen concentrations at which the FLASH effect occurs, disappearing at 0% dissolved oxygen, as well as at normoxia (about 20% oxygen), reaching a maximum at a concentration of around 0.25–1.5% [108,139,140]. Of course, complete radiation depletion of oxygen would lead to partial irradiation of samples in a state of anoxia, which is gentler on macromolecules. Further studies have shown the significant difference in radiation depletion of oxygen when comparing different irradiation modes. Although computer simulations of proton irradiation in FLASH mode showed significant depletion of molecular oxygen during irradiation in the range from 1 ns to 10 μs, which the authors attributed to the sparing of normal tissues during FLASH irradiation [141], direct measurements showed (Table 2) that less oxygen was consumed at higher dose rates than at standard dose rates. However, complete depletion of oxygen for photons, protons, and carbon ions in water samples irradiated in FLASH mode (10 Gy) was not observed [95]. Oxygen depletion in irradiated albumin solution [10,96] or in cancer (HAP1) and normal (HEK-293T) cell lines [99] was primarily dose-dependent, not mode-dependent. For every 20 Gy drop in oxygen concentration, the reduction in oxygen depletion upon reaching FLASH was an additional 20% of the initial 5% total oxygen level. These are clearly very small amounts, insufficient to create local hypoxia and to explain the bulk of the observed FLASH effect by oxygen depletion. At the same time, a number of authors believe that radiation-induced transformations in the cell involving oxygen are still poorly understood, which requires more in-depth research into how oxygen can be involved in the mechanisms of the FLASH effect [99].

Interestingly, although the FLASH effect has been demonstrated for most types of IR used in radiobiology, for some particles, ROS generation was independent of oxygen partial pressure when irradiated with accelerated carbon ions under hypoxic conditions (<0.5% O_2_). When irradiated with X-rays or carbon ions at a dose of 64 Gy, H_2_O_2_ production decreased. However, higher-LET carbon ions were able to generate more H_2_O_2_ in an O_2_-independent manner than lower-LET carbon ions or X-rays [132]. In vivo, a number of low-molecular-weight oxygen-containing inorganic compounds can influence ROS production: CO_2_, like O_2_, can increase H_2_O_2_ production and e_aq_^−^ scavengers such as N_2_O and NaNO_3_ can significantly reduce the difference in H_2_O_2_ levels recorded at ultra-high and standard dose rates [133]. It should also be noted that living cells contain enzymes capable of regenerating oxygen [142]. The hypothesis of radiation-induced oxygen depletion or reduced ROS generation should also be considered in the context of antioxidants, which can significantly contribute to the reduction in ROS levels at different oxygen concentrations [70,111,125,127].

On the other hand, at certain concentrations, hydrogen peroxide can also act as a signaling molecule, triggering the transformation of cellular processes and thereby influencing the radiation response [143,144,145]. When assessing radioprotection in spheroids with different levels of oxygenation of cell layers at different distances from the surface, a radioprotective FLASH effect (according to the criteria of ATM/DNA-PKcs activation in radiation-induced DNA damage) was found only for well-oxygenated (3%) outer layers of the spheroid when irradiation was carried out at 37 °C. However, after irradiation at 4 °C, radioprotection was observed throughout the entire spheroid volume [104]. At the same time, in earlier studies on 2-dimensional cell cultures, it was shown that the maximum radioprotective FLASH effect according to the survival criterion was achieved under hypoxic conditions (1.6%) [139]. A comparison of the findings of these two studies suggests that inside the spheroids at 37 °C, not only oxygen starvation but also general metabolic starvation may occur, which does not allow for reparative kinases (monitored markers: pDNA-PK_Ser2056_, pH2AX_Ser139_—products of the work of such kinases) to work at full strength, which may hide the true difference between the modes. At the same time, a detailed analysis of damage to eukaryotic genomic DNA analogs—supercoiled pBR322 plasmids—irradiated with a proton beam at doses ≤ 10 Gy under FLASH and CONV conditions at different oxygen tensions, absorber levels, pH, and Fe(II) concentrations showed that plasmid DNA strand breaks are independent of the dose rate at doses below 10 Gy [146]. When modeling FLASH-RT for the lungs, it is necessary to resolve a certain difficulty associated with the location of pulmonary epithelial cells at the interface between two phases—air (21% oxygen) and water (tissue oxygen level is significantly lower). When comparing the FLASH mode with the CONV mode during irradiation of donor human lung endothelial material (based on biopsies from 2 donors) in an air environment, the regenerative potential of the cells significantly decreased after 7 days, compared to 1 day after irradiation in the case of seeding [147]. This indicates the absence of radioprotective FLASH effect in this case, albeit with the caveat that the dose was only 2 Gy, which is generally insufficient to register the FLASH effect. It is worth mentioning here the data on carbon ion irradiation with a dose rate of 1.6 Gy/s (CONV) or 100 Gy/s (FLASH) of human salivary gland cells (HSGc-c5), human dermal fibroblasts (HDFs), and human bronchial epithelial lung cells (Nuli-1) under physioxia and at an atmospheric oxygen level of 20% [105]. In this case, radioprotection was detected only under mammalian normoxia (4%), and not at 20%, which is closer to the conditions in the study [147]. In this case, the classical FLASH effect, observed in many other publications under hypoxia-physioxia, is not refuted. Probably, for a better understanding of the role of oxygen concentration in the FLASH effect for lung tissue, the blood flow rate against the background of the diffusion of oxygen and carbon dioxide during gas exchange will also have a certain significance.

Obtaining more accurate information about radiation-induced oxygen depletion is possible through the use of cell- and tissue-compatible sensors for monitoring oxygen concentration in real time. Pétusseau et al. measured tissue oxygenation by assessing endogenously produced delayed porphyrin fluorescence signals [103]. 5-Aminolevulinic acid was used as a precontrast agent and was administered intraperitoneally to mice bearing tumors (BxPC3 human pancreatic cancer cells). The precontrast agent was metabolized in most tissues into a red fluorescent molecule, protoporphyrin IX (PpIX). PpIX exhibits both fast fluorescence, indicating oxygen concentration, and delayed fluorescence, which is enhanced in situations of low tissue oxygen levels [103]. Using this method, it was shown that intracellular and extracellular tissue oxygenation observed during ultra-high dose rate radiation therapy (10 MeV electrons in 3 μs pulses at 360 Hz with doses of 10, 22, and 28 Gy) in mice bearing tumors from five different cultured cancer cell lines were consistent with those quantified by measuring extracellular oxygen [45].

Oxygen consumption in tissues depends on the type of tissue and its oxygenation, which, for example, was shown when irradiating mouse skin (19.8 ± 0.3 Gy) with electrons in the Mobetron linear accelerator with an ultra-high dose rate [148]. Dose-dependent local oxygen depletion was detected in tissues (tumor tissue, skin, muscle, and brain) for the FLASH mode, but not for the CONV mode: intracellular oxygen (Oxyphor PtG4) levels were measured in vivo in tumor (mice bearing subcutaneous human glioblastoma U-87 MG tumors) and normal mouse tissues (skin, muscle, and brain) during electron irradiation (6 MeV electron linear accelerator) in FLASH (1300 Gy/s) and CONV (2 Gy/s) modes in the dose range of 10–40 Gy. The authors note that variability in oxygenation levels was observed among individual animals for tumor, skin, and muscle tissues [21].

When local irradiation of the limbs of mice with intramuscular syngeneic fibrosarcoma *LSL-KRAS^G12D/wt^*; *p53^FL/F^* [97] or 20 Gy of the limbs of mice with subcutaneously injected xenograft MDA-MB-231 [10] was performed, a decrease in oxygen concentration in vivo was observed in the irradiated area (accordingly, the determination was carried out either with phosphorescent sensors Oxyphor PtG4, administered intramuscularly, or Oxyphor 2P, administered intravenously). This decrease in oxygen concentration was at a level comparable to in vitro tests only for the FLASH mode, while for the CONV mode no change in oxygenation of either tumor or normal tissue was observed. The absence of a decrease in oxygenation detected by the phosphorescent sensor after CONV irradiation is in good agreement with the significantly lower (by several orders of magnitude, depending on the final dose rate of the FLASH mode) irradiated blood volume in the case of a conditionally instantaneous dose delivery [149]. Further development of technologies for a high-resolution in-tissue oxygenation measurement are needed in both normal and tumor tissues, as oxygen levels vary significantly, ranging from tens of micrometers [150].

Interestingly, in tumor grafts that were initially hypoxic, the FLASH mode-induced radiation depletion of oxygen was reduced by 2–3 times [10,97,151], while in more normoxic U-87 MG GBM cancer transplants, the kinetics of oxygen depletion did not differ from that for healthy normoxic tissues [21].

Thus, the tendency to preserve normal tissues over cancerous tissues [2], based on the hypothesis of radiation-induced oxygen depletion [152], may be more truthful for hypoxic tumors or hypoxic regions of tumor. However, much more relevant data about hypoxic subregions of tumor, especially poor vascularizated central highly hypoxic regions in comparison with better oxygenated periphery are needed to make a strong statement for this case. The degree of oxygenation/hypoxia of tumor tissue can vary. The heterogeneous structure of microcirculation in the tumor can have a significant impact on the occurrence of hypoxic microregions [94,153]. The experimentally measured oxygen diffusion distance in tumor tissue ranges from 100 to 200 μm [154,155]. Furthermore, tumor hypoxia can vary significantly over time, within hours or days [156,157]. In this regard, it is very important to evaluate changes in tissue oxygen levels induced by treatment procedures online.

When studying the influence of irradiation on the red-ox metabolism of tumors, it is important to take into account the direct link between regular cellular mechanism-producing ROS, pathological ROS overproduction proper to tumor metabolism, and mitochondrial dysfunction induced by ionizing radiation in normal cells, which leads to an increase in ROS in them [158]. The baseline level of ROS in tumor cells is higher than in normal cells due to an imbalance between oxidants and antioxidants [159]. Radiation exposure increases ROS levels. FLASH irradiation is believed to generate higher levels of ROS in tumor tissue [158]. On the other hand, by briefly disrupting mitochondrial function in normal cells, FLASH irradiation reduces mitochondrial ROS production, thereby increasing, for example, the radio-resistance of FLASH-irradiated mouse embryonic fibroblasts or normal human lung fibroblasts IMR90 [76,160].

Since the vast majority of animal irradiation experiments are performed under anesthesia, and anesthesiological practices vary greatly between research groups, it is important to consider the method of anesthesia used, which may result in differences in tissue oxygenation. Carbogenic inhalation (5% CO_2_ and 95% O_2_) combined with isoflurane during irradiation halved FLASH radioprotection as measured by cognitive function preservation in animals [70]. Inhalation of 70% oxygen combined with isoflurane reduced the rate of skin ulcer regeneration after irradiation in the FLASH mode, while no significant change in the severity of skin reactions was observed for the CONV mode [6]. Interestingly, the effects of anesthesia depended on the sex of the experimental animals. Varying the oxygenation level using 100% oxygen as an anesthetic carrier gas revealed sex-dependent differences in tissue oxygenation in male and female C57BL/6 mice, resulting in different responses to FLASH irradiation: the sparing FLASH effect was more reduced in females than in males under 100% oxygen [38]. Mice irradiated with ultra-high dose rate electrons (Mobetron) and breathing room air showed a mean time to skin ulcer formation of more than 20 days, which is significantly longer than 9.5 days at a conventional dose rate. No significant difference in ulcer formation time was found between male and female mice irradiated with FLASH mode and receiving room air. Clearly, both tissue oxygenation and gender are likely sources of variability during ultra-high dose rate irradiation [38]. In some experiments [100], mice (C57BL/6) were anesthetized with gas mixtures containing isoflurane in different percentages or ketamine/xylazine (ket/xyl: 100/10 mg/kg). Oxygen tension (pO_2_) was measured in the skin with Oxyphor PdG4 and inside cells with protoporphyrin IX (PpIX). Under all isoflurane anesthetic conditions, leg skin pO_2_ levels increased significantly and reliably from 12 to 15 mmHg at the beginning of anesthesia induction (4–6 min) to 24–27 mmHg after 10 min. Ketamine/xylazine anesthesia resulted in skin pO_2_ maintenance at 15–16 mmHg over the 10 min study period. An increase in pO_2_ in mice breathing isoflurane was demonstrated using Oxyphor and delayed fluorescence assays of PpIX, indicating similar intracellular and extracellular oxygenation. The authors emphasize that the timing between anesthesia induction and radiation may be critical to minimize variability in radiation response [100]. While FLASH could seriously change local concentration of oxygen it is important to note that some details influence anesthesia gas mixtures; effect on general tissue oxygenation. Inhalation of carbogen or 100% O_2_ increased O_2_ levels in the cerebral cortex of mice by 0.28 and 0.26 mmol/L, respectively. O_2_ molecules readily cross the blood–brain barrier and are rapidly distributed throughout brain tissue. Inhalation of carbogen results in faster O_2_ distribution throughout brain tissue than inhalation of 100% O_2_ [161]. Inhalation of 100% O_2_ has been shown to cause vasoconstriction, whereas inhalation of 5% carbon dioxide in carbogen gas caused vasodilation, which increased blood flow [162]. Therefore, the rate of O_2_ distribution may be decreased by inhalation of 100% O_2_ and increased by inhalation of carbogen [161].

### 2.3. Cooperative Response

An interesting hypothesis of less radiation damage to healthy tissues, which is observed during irradiation of a tumor graft [2], stems from the first two (the hypothesis of differential water radiolysis and oxygen depletion) together with the consideration of the cooperative response of the organism to irradiation or the abscopal effect in response to the FLASH regimen. Indeed, the case of the FLASH-mode irradiated volume is a few magnitudes lower than in the case of CONV mode [149]. Thus, local irradiation of an animal model in vivo in FLASH mode can be extrapolated to the results obtained in vitro, assuming that most radiation damage is localized strictly in the irradiated area. Moreover, relatively long-lived radiolysis products, such as hydrogen peroxide, are localized predominantly in the immediate vicinity of the irradiated area. As well intracellular content separated by the cytoplasmic membrane, extracellular liquid (first of all blood and less-lympha, etc.) shows radical-radical self-annihilation. Such a reduction in the level of ROS is well characterized by a closed water container irradiation model. In the case of local radiation exposure to an animal model in CONV mode, the accumulation of ROS are better described by a closed water container irradiation model as well as by a flow-through cell radiolysis model. Therefore, the first model better describes processes in intracellular liquid phase, the second “flow through” model better suits for extracellular biological liquids, especially for active circulating blood and the lympha [134]. On its turn-flow chamber leads the stable outflow of radiolysis products from the irradiated area, which leads to significantly larger accumulation of quasi-stable water radiolysis products within the rest of the whole animal. Even if quasi-stable ROS are distributed with less total concentration, the greater total amount of them may be enough to activate hyperinflammation, that will, in turn cause severe radiotoxicity—a common feature of FLASH mode (Table 4).

Indeed, the activation of cooperative immunology generally needs to meet the threshold of a stimulus of a certain amplitude. That is, one in good agreement with the observation that FLASH mode-driven reduction for normal tissue needs to be induced by dose of a few Gy, usually about 10 Gy for local irradiation or even much more (Table 4). The radiotoxicity after FLASH irradiation progressively reduces as the dose increases until it reaches the body’s breaking point, which may happen when the dose is too high for the tissue to show a difference in reaction and when total irreversible destruction of the organism occurs. A row of indirect evidence supports this hypothesis. For example, through the increase after the CONV regime of inflammatory factors spreading with the blood flow—the cytoplasmic fraction of DNA and the HMGB1 protein [57,69]—as well as through the influence of hydrogen peroxide functions as a mediator of signal transmission of redox metabolism and the immune response to damage [143,144,145]. Groups of immunological evidence indirectly support the role of transferring water radiolysis products and the damage associated molecular pattern species across the whole organism. The hyperactivation of irradiation-induced inflammation and pyroptosis via the cGAS-STING axis and subsequent animal death was only shown for the CONV-irradiated group in the case of a proinflammatory immunological genetic background PD-1/PD-L1-KO [57]. According to scRNA-seq and flow cytometry data, FLASH mode activates microglia less with the participation of the INF-I system [14]; it activates proinflammatory Ccrl2+ neutrophils and Mefv^+^ monocyte less, and activates immunosuppressive CD4+ CD40L+ T-helpers and mediated TGF-β signaling and epithelial–mesenchymal transitions in alveolar type I cells more [40]. Reduced radiational toxicity by FLASH mode is dramatically decreased in the case of hypofractionation with pauses in irradiation higher than 15 s [101] (minimal pause duration—0.1 s) or 2 min [163] (minimal pause duration 2 min). This also corresponds to the re-oxygenation of an irradiated area as well as an increase in quasi-stable ROS flowing out of the irradiated zone, which is accompanied by an increase in their total amount in the whole organism. Knowing that the integral biological response of partly irradiated animals in FLASH mode together with data about the direct increase in viability of cultured cells irradiated in FLASH in a vial, where the amplitude of radioprotection after FLASH mode dramatically increased for higher doses [77,140,164], especially in case of hypoxia [140], and showed less senescence with less TGF-β expression [72], let consider that the FLASH effect is much more complex. So for partly irradiated healthy animal tissues, FLASH sparing may first of all be a product of the cooperative response of the whole organism where the communication between irradiated and unirradiated parts is crucial. For the in vitro study, with fully irradiated objects such as cells or whole embryos, the main mechanism may be linked with the self-annihilation of free radicals. That is why an in vitro FLASH investigation showed increase in viability for both cancer and normal cells in the vast majority of cases, whereas for animal cancer grafts irradiated enough to kill the majority of susceptible to IR cells only for healthy tissues, FLASH-mode radioprotection emerged. However, further research is needed.

### 2.4. The Role of Iron in the Metabolism of Reactive Oxygen Species in the FLASH Effect

Unlike the highly reactive OH radical, which reacts with the first molecule encountered, hydrogen peroxide has a long lifetime and is relatively stable in the absence of transition metal ions. Being an electrostatically neutral molecule, it can diffuse from the site of formation and easily penetrates cell membranes, causing damage to cellular organelles at relatively high concentrations. In the presence of iron or copper ions in cells and tissues, hydrogen peroxide can produce cytotoxic OH^•^ radicals in the Fenton reaction or in a Fenton-like reaction at a physiological pH [11,106]. Cancer cells are prone to iron accumulation and may thus be more susceptible to death in response to peroxide accumulation than normal cells [165]. The next hypothesis, previously put forward as an explanation for the difference in the response of tumor and normal tissues to the FLASH mode [2], directly related to the level of oxygen and ROS, is the assumption of different levels of iron metabolism and the associated processing of ROS, primarily hydrogen peroxide in Fenton-like chemistry reactions. Although a significant array of data on the correlation between iron concentrations in tumor cells and grafts has not yet been collected, the first experimental confirmation of increased lipid peroxidation and loss of radioprotective properties of FLASH irradiation in intestinal crypts in the case of an iron-rich diet has appeared [11]. At the same time, the hypothesis of the dependence of the ferroptosis level on the dose delivery mode during the irradiation of stable cell lines A549 and MDA-MB-231 was not confirmed [11]. Considering that significantly higher iron concentrations could be achieved in the intestine due to diet than in the intracellular space, these studies speak against the hypothesis of the role of ferroptosis mediated by the differentiated metabolism of tumor and normal cells.

### 2.5. Anoxic Structural Changes in Macromolecules in Response to Irradiation in FLASH Mode

Another hypothesis explaining the cause of FLASH mode was the probable radiation-induced recombination of radicals in organic macromolecules, resulting in the synthesis of hybrid macromolecules that are potentially more dangerous for tumor cells with impaired homeostasis systems. To date, several studies have analyzed macromolecular structures, but they have not demonstrated the formation of covalent crosslinks between DNA in vivo [166] or the formation of crosslinks between oligopeptides in vitro [117]. However, in vivo infrared microspectrometry has shown that irradiation in FLASH mode results in less disruption of the conformational spectrum of key cellular and tissue macromolecules: DNA, proteins, and lipids [44]. Overall, conformational changes in the three-dimensional structures of proteins, their hydration shells, hydrophobic and electrostatic interactions, and, ultimately, their functions in fast radiolytic processes under FLASH-mode conditions remain largely unexplored.

Based on the presented model experiments, it is clear that the key features distinguishing FLASH mode from irradiation with a conventional dose rate are reduced oxygen consumption and the yield of water-stable ROS, in particular hydrogen peroxide and possibly peroxides of biological molecules, which varies depending on the time interval after irradiation and the composition of the irradiated mixture.

## 3. Biological Aspects of the FLASH Effect on the Cellular, Tissue, and Organismal Levels When Irradiated with IR Sources with Different LETs

### 3.1. Electrons

A large number of studies on the FLASH effect have been carried out using electron irradiation of biological models, as hadron therapy systems are rarer and more expensive to use [84]. Compared with the CONV mode, the electron irradiation of intact mouse skin in the FLASH mode (clinical linear accelerator, 10 MeV, 270 Gy/s for FLASH-RT and 9 MeV, 0.12 Gy/s for CONV-RT) resulted in a 7-day prolongation of the skin toxicity latency (to moist skin desquamation) after a single dose of 25 Gy, but not after 30 Gy, where no differences were observed between FLASH-RT and CONV-RT. The histomorphological characteristics of skin damage were similar for FLASH-RT and CONV-RT. An evaluation of the treatment efficacy (time to tripling of tumor volume) of GL261 murine glioma and B16F10 murine melanoma cells transplanted into mice irradiated with electrons at doses of 1 × 11 Gy, 1 × 15 Gy, 1 × 25 Gy, 3 × 6 Gy, and 3 × 8 Gy revealed no difference between FLASH-RT and CONV-RT. For B16F10 cells, FLASH-RT resulted in comparatively high changes in the expression levels of genes involved in angiogenesis and vascular maintenance. Quantification of B16F10 cell counts for CD8+ and CD4+ markers revealed no difference between FLASH-RT and CONV-RT in any dose group except for the 1 × 11 Gy FLASH group [13]. Similar results for electron-induced skin toxicity (16 MeV) were obtained at higher doses of 30 and 40 Gy: single-fraction hemithoracic electron irradiation of C57BL/6 mice skin in FLASH mode resulted in both a lower incidence and a lower severity of skin lesions (as measured by ulceration) after 8 weeks compared to CONV-RT. The beam parameters for FLASH-RT were as follows: pulse repetition rate of 90 Hz, dose per pulse of 2.0 Gy, average dose rate per fraction of 180 Gy/s, and instantaneous dose rate (in a 5 μs pulse) of 4.0 × 10^5^ Gy/s. Survival was also higher after FLASH-RT of hemithoracic irradiation (survival >180 days at doses of 30 and 40 Gy) compared with CONV-RT (survival 100 and 52 days at 30 and 40 Gy, respectively). Ulcers were not observed at doses of 20 Gy or lower in either FLASH-RT or CONV-RT. These results suggest a rightward shift in the dose–response curve for radiation-induced skin ulcers for FLASH-RT compared to CONV-RT, indicating significant potential for improving the therapeutic index in radiotherapy [167]. Using another model, intracranially implanted NS1 glioblastoma (rats, syngeneic), it was shown that after head irradiation with an electron beam (10 MeV clinical linear accelerator, Elekta Precise, Stockholm, Sweden), the dose–response relationship for animal survival was similar for CONV-RT and FLASH-RT at 30 Gy and did not differ from the control (mice with tumors without irradiation), despite a decrease in tumor size. However, at lower FLASH-RT doses (20 and 25 Gy), survival was higher than in non-irradiated tumor-bearing mice. Moreover, tumor size was inversely proportional to the radiation dose during the 30-day post-irradiation period. The authors believe that the 30 Gy radiation dose was too high compared to the acute radiotoxic effects that the animals could tolerate [4]. To identify the specific effects of irradiation using different accelerators, the results of electron irradiation conducted at two institutions were compared: Stanford University (California, USA) and the University of Lausanne (CHUV—Centre Hospitalier Universitaire Vaudois, Switzerland). Irradiation at Stanford was carried out using a Varian medical linear accelerator. Irradiation in Lausanne was carried out using a prototype Oriatron6e electron linear accelerator. Adult tumor-free female mice received a whole-brain dose of 10 Gy for FLASH-RT or CONV-RT. The results obtained with both devices were very similar: after 3 weeks, greater recovery of immature neurons, as assessed by doublecortin (DCX) staining, was observed with FLASH-RT than with CONV-RT [39]. In contrast to the described response of immature neurons to electron exposure, in mature and dendritic subsets of neurons located in the pyramidal layer of CA1 and the prelimbic/infralimbic region of the medial prefrontal cortex, the dependence of morphological parameters on the dose rate during electron irradiation of the head of mice at a dose of 10 Gy was not detected in CA1 and mPFC neurons, which turned out to be more radioresistant [43]. A significant increase in the volume of activated microglia was observed in both CONV and FLASH cohorts compared to non-irradiated controls: double staining of IBA-1 (microglial staining) and CD68 (activated microglial staining) cells was performed in the mouse hippocampus 48 h after irradiation [39]. Infrared scanning raster microspectroscopy (IRSMS) maps of mouse brain sections were collected 24 h after electron irradiation (eRT6-Oriatron) of C57Bl/6J mouse brains using FLASH-RT (1.8 μs pulse) and CONV-RT (0.1 Gy/s) at the same dose of 10 Gy. Analysis of several brain regions revealed that differences in FLASH-RT and CONV-RT images are associated with protein modifications, nucleic acid fragmentation/condensation, and lipid changes. Fine molecular modifications of several functional groups associated with proteins, nucleic acids, carbohydrates, and lipids, detected simultaneously, revealed a greater agreement between FLASH-RT and control compared to CONV-RT [44]. At the cellular level, a decrease in oxylipid production was shown as early as 5 min after FLASH irradiation of normal normoxic cells [168].

To investigate the immune response as a potentially important mechanism of the antitumor FLASH effect, various mouse tumor models were transplanted either subcutaneously or orthotopically into immunocompetent (C57BL/6J) mice and moderately (NU[Ico]-*Foxn1^nu^*) or severely (*NOD-Rag1^null^ IL2rg^null^*) immunodeficient mice, where as a first approximation, no difference in antitumor efficacy between the regimens was found based on the post-radiation growth rate of the inoculated tumor [20].

Local electron beam irradiation (eRT6 Oriatron, PMB-Alcen) with a single dose of 20 Gy or fractions (3 × 8 or 2 × 6 Gy) was performed in the FLASH (≥2000 Gy/s) and CONV (0.1 Gy/s) modes. FLASH and CONV modes were found to be equally effective in delaying tumor growth in immunocompetent and moderately immunodeficient mice, both with single and fractionated irradiation. Interestingly, in mice with severe immunodeficiency, the FLASH mode retained antitumor activity compared to controls, suggesting a possible antitumor mechanism independent of the adaptive immune response lost in the immunodeficient mouse model. Furthermore, FLASH and CONV modes did not increase transforming growth factor-β1 levels in tumors compared to non-irradiated animals. Both irradiation modes were capable of inducing a long-term immunological memory response. These results clearly indicate that tumor responses in several immunocompetent and immunodeficient mouse models are largely independent of dose rate [20].

A more precise understanding of the role of immunity in the post-irradiation response has become possible with the advent of more in-depth analysis of immune cells using single-cell RNA sequencing. Single-cell RNA sequencing (scRNA-seq) and flow cytometry analysis of immune cells isolated from an orthotopic syngeneic mouse model of diffuse midline brainstem glioma after tumor irradiation with electrons (Varian Clinac 2100C linear electron accelerator) at a dose of 15 Gy in FLASH (90 Gy/sec) or CONV (2 Gy/min) modes revealed time-varying (4 and 10 days post-irradiation) ratios in immune cellular compartments. Although tumor control and survival were similar between CONV and FLASH, the radiation cellular response depended on the cell type and dose rate. While the effects induced by FLASH and CONV-RT did not differ on day 4, a significant increase in the interferon receptor type 1 α/β was observed in the microglial cell population on day 10 with CONV-RT and control compared to FLASH-RT. In non-resident myeloid clusters of macrophages and dendritic cells, an increased enrichment of the interferon pathway type 1 was found with CONV compared to FLASH and control [14]. Following 18 Gy of 6 MeV electron beam irradiation, single-cell sequencing (sc-RNA-seq) demonstrated reduced activation of factors that mediate prolonged post-radiation inflammation, particularly reduced activation of Ccrl2+ neutrophils and Mefv^+^ monocyte signaling, which inhibits excessive activation of proinflammatory macrophages. FLASH-RT also elicits stronger activation of CD4+ CD40L+ T-helpers, thereby reducing inflammation, and activates TGF-β signaling and the epithelial–mesenchymal transition in alveolar type I cells, accelerating regeneration and resulting in fewer side effects from radiation by day 7 [40]. Using various subcutaneous tumor models in mice (glioblastoma, head and neck cancer, and lung adenocarcinoma cells) irradiated with an electron beam (Oriatron6e linear electron accelerator, 6 MeV) at a dose of 20 Gy, it was found that the FLASH mode (100 Gy/s) maintained the antitumor efficacy of irradiation under acute hypoxia, unlike the CONV mode. Subsequent molecular analysis using RNA sequencing (RNAseq) revealed FLASH-RT-specific changes in human glioblastoma cells: cell cycle arrest, reduced ribosome biogenesis, and a switch from oxidative phosphorylation to glycolysis. The inhibition of glycolysis with trametinib increased the efficacy of FLASH-RT under both normal and hypoxic conditions mediated by mechanical compression of the tissue area. These results indicate that, in addition to the normal tissue-sparing FLASH effect, this irradiation mode can overcome hypoxia-mediated tumor radioresistance by specifically switching metabolism to glycolysis [22]. Subsequent research maybe targeted to the analysis of how shifts in oxphos energy methabolism to glycolysis, which directly drives oncogenesis via energy and building blocks supply as well as extracellular matrix reorganization [169,170] and how this could also be used in complex anticancer strategies. Work to search for other differentially expressed genes in normal human fibroblasts after a dose of 10 Gy gives hope for the discovery of biomarkers explaining the FLASH effect for its more effective practical applications [171]. Less lung tissue damage and a lower level of pulmonary fibrosis in C57BL/6J mice in the FLASH (200 Gy/s) group compared to CONV (0.3 Gy/s) were detected at a late time point—3 months after whole-body electron beam irradiation of mice with a dose of 3 Gy. In that study, the first proteomic sequencing (4D-Fast DIA) was performed for the radiobiological study of the FLASH effect. It revealed significant differences in CCT6b protein expression between the two irradiation groups. The authors suggest that the differential response in pulmonary fibrosis levels induced by the two types of radiation may be related to the expression level of CCT6b protein, but the specific mechanism of action requires further study [172].

### 3.2. Photons

The first experiments to identify the FLASH effect using ultra-high-dose X-rays (1000 Gy/s) were conducted by a group of researchers to control tumors (breast cancer cells injected subcutaneously), damage to normal lung tissue (chest irradiation), and bowel tissue (abdominal irradiation). A reduction in tumor size and a significant increase in survival in tumor-bearing patients were found with FLASH-RT and CONV-RT. In mice irradiated abdominally or thoracically, longer survival was observed with FLASH-RT compared to CONV-RT [30]. Using an X-ray laser, FLASH mode was shown to suppress the development of radiation-induced pyroptosis by reducing the activation of the cGAS-STING signaling cascade and decreasing the accumulation of the cytosolic DNA fraction. This finding may be useful in the development of combined radio- and immunotherapy [57]. In particular, FLASH-RT was found to induce various immune responses in the spleen and reduce radiation-induced damage to the spleen and intestine. Although both FLASH-RT and CONV-RT (10 Gy, abdominal irradiation) effectively suppressed the growth of subcutaneously injected Py8119 and Py230 breast tumor cells, FLASH-RT reduced the level of splenomegaly and led to an increase in the level of CD8+ T cells and a decrease in the level of CD4+ T cells in the splenic microenvironment compared with CONV-RT in C57BL/6 mice. Furthermore, FLASH-RT reduced tissue damage and inflammatory response in the small intestine compared with CONV-RT [19]. The FLASH effect was also observed when irradiating the mouse chest at a dose of 30 Gy using 10 MV photon irradiation for CONV or FLASH-RT, based on calculations of functional residual capacity and tidal volume [173].

### 3.3. Protons

The real advantage of protons and heavy ions over electrons and photons is the ability to utilize the spread-out Bragg peak (SOBP), which allows for high-dose delivery to the target with a lower dose to nearby healthy tissue. An additional advantage of protons over heavier ions, such as accelerated carbon nuclei, is the almost complete particle arrest at the Bragg peak, whereas for carbon ions, approximately 30% of the energy is deposited in the region after the Bragg peak [83,84,174]. Furthermore, compared to light particles, protons allow for the use of high-LET irradiation, especially in the Bragg peak region, and their beams are less blurred when passing through tissue.

A number of authors have compared identical biological models using electron and proton systems in a single publication. When comparing the novel object recognition performance of electron (e) and proton (p) beams in FLASH (eFLASH and pFLASH) and conventional (eCONV and pCONV, respectively) modes, mice irradiated to the head with 10 Gy of eFLASH and pFLASH modes demonstrated statistically indistinguishable object recognition rates compared to the control group, whereas mice irradiated in eCONV and pCONV modes demonstrated deterioration in the measured performance compared to the control group. The eRT6/Oriatron/CHUV/5.5 MeV electron accelerator and the Gantry1/PSI/170 MeV proton gantry were used to deliver doses in conventional (0.1 Gy/s eCONV and pCONV) and FLASH (≥110 Gy/s eFLASH and pFLASH) modes. Furthermore, tumor radiation response (GL261 cells implanted in the flank of mice) was similar between eFLASH and pFLASH compared to eCONV and pCONV [12].

Proton irradiation (SNAKE ion microprobe, 20 MeV) at doses of 23 or 33 Gy to the ear of Balb/c mice using three dose rates (CONV = 0.06 Gy/s, FLASH9 = 9.3 Gy/s and FLASH930 = 930 Gy/s) revealed a protective FLASH effect in terms of a reduction in ear inflammation and swelling for a dose of 33 Gy for dose rates of 9 and 930 Gy/s compared to the CONV dose rate. An assessment of the volume of irradiated blood shows that using the CONV mode irradiates 100 times more blood than using the FLASH9 or FLASH930 modes, indicating a significant role of blood in the primary mechanisms of the protective FLASH effect [149].

Irradiation of the head and neck often damages the salivary glands, causing their dysfunction. C57BL/6 mice with MOC2-luc cells implanted in the lateral tongue (an orthotopic head and neck tumor model) were irradiated to the head and neck region with protons (IBA Proteus Plus Cyclotron, 230 MeV) either as a single dose of 14–18 Gy or 8 × 3 Gy in FLASH (128 Gy/sec) or CONV (0.95 Gy/sec) modes. FLASH-RT was found to reduce normal tissue toxicity, as measured by overall survival and improved salivary flow without compromising tumor control; mice irradiated in the CONV-RT mode exhibited increased fibrosis of the tongue epithelium [29].

Thoracic irradiation may also expose the heart to radiation. In mice irradiated in the FLASH mode with protons (IBA Proteus Plus Cyclotron, 230 MeV) in the apex of the heart with a single dose of 40 Gy, acute and chronic toxicity were reduced with lower expression of inflammatory cytokines and profibrotic factors in the post-radiation period compared to CONV-RT [175].

According to the gastrointestinal toxicity criterion in C57BL/6J mice irradiated abdominally with protons (87 MeV synchrotron) in SOBP (230 Gy/s—pFLASH; 0.3 Gy/s—pCONV) and at plateau (150 Gy/s—pFLASH; 0.2 Gy/s—pCONV) or electrons (linear accelerator; eCONV—0.4 Gy/s, eFLASH—188–205 Gy/s) at similar doses of 12–14 Gy, a negative proton-specific FLASH effect was revealed for protons compared to electron irradiation: the number of regenerating crypts in the small intestine was the lowest for pFLASH regardless of the beam configuration and the highest for eFLASH [56]. Whole-abdominal irradiation of mice with 14 Gy protons (IBA Proteus Plus Cyclotron, 230 MeV) in CONV and FLASH modes (0.82 ± 0.1 Gy/s, 125.3 ± 4.8 Gy/s, respectively), revealed faster weight recovery and increased lifespan in FLASH-mode irradiated mice for approximately 300 days post-irradiation compared to CONV-RT. Accelerated differentiation of stem cells (revSCs) essential for intestinal regeneration, as well as qualitative and quantitative changes in the activity of signaling pathways important for revSC differentiation and epithelial regeneration were observed. Specifically, FLASH-RT resulted in increased intestinal tissue infiltration by macrophages producing TGF-β, a factor required for revSC induction, which was associated with enhanced TGF-β signaling in revSC. In pericryptal fibroblasts, FLASH-RT resulted in increased type I interferon (IFN-I) signaling, which directly stimulates the production of FGF growth factors that support revSC proliferation. Accordingly, the ability of FLASH to improve epithelial regeneration depended on IFN-I signaling. However, in the context of conventional radiation, IFN-I had a detrimental effect and contributed to radiation toxicity. Thus, a tissue-level signaling network coordinated by differences in IFN-I signaling and involving stromal cells, immune cells, and revSCs underlies the ability of FLASH-RT to reduce normal tissue toxicity without compromising antitumor efficacy in this model [50,176]. In contrast to these results, when using different equipment (Hitachi synchrotron: HITACHI, Ltd., Tokyo, Japan, 87 MeV), a significant deterioration in the survival of mice irradiated with protons in the FLASH mode was shown compared to the CONV mode 50 days after irradiation of the whole abdomen of mice at comparable doses of 12–14 Gy. Irradiation was performed in the FLASH mode in SOBP (230 Gy/s) or plateau (150 Gy/s), or in the CONV mode (0.3 Gy/s, 0.2 Gy/s, respectively). Furthermore, artificial intelligence-based crypt analyses (using crypt number per cm of tissue or crypt depth between groups) performed 4 days after irradiation revealed no significant differences in these parameters between the irradiation regimens used [53].

A study of irradiated human epithelial cell organoids obtained from donors showed no significant differences in DNA damage or gene expression profiles (*MDM2*, DDB2, *AEN*, BAX, *P21*, *GDF15*, and *MKI67*) between the CONV and FLASH modes at doses of 2 and 8 Gy at 7 and 24 h. More complex models with alveolar, endothelial, and immune cells could provide a deeper understanding of the FLASH effect [147].

Using a genetically modified mouse model of medulloblastoma, FLASH-RT was found to stimulate proinflammatory polarization in tumor macrophages after irradiation with accelerated protons [27], which could be used to develop clinical applications of FLASH-RT for this extremely difficult-to-treat disease.

Conflicting results were obtained for other tissues. Although FLASH-RT of the abdominal region of C57BL/6J mice with a proton beam at a dose of 15 Gy in SOBP promoted the preservation and regeneration of intestinal crypts compared with CONV-RT at 3.5 days post-irradiation [176], irradiation of the abdomen of similar C57BL/6J mice with pencil beam proton at a dose of 14 Gy (250 MeV; 0.6 Gy/s for CONV and 80–100 Gy/s for FLASH) showed a significant impairment of survival after FLASH-RT compared with CONV-RT, with no apparent difference in intestinal histology at 4 days post-irradiation [53].

### 3.4. Heavy Ions

Using cell models—B16F10 murine melanoma and Pan02 murine pancreatic adenocarcinoma—irradiated with carbon ions at Osaka University (Osaka HIMAK) at a LET of 50 keV/μm and dose rates of FLASH (380 Gy/s) and CONV (1.16 Gy/s) modes—we assessed the survival and expression of immunologically related proteins. Taken together, FLASH-RT with carbon ions demonstrated a potentially greater effect on the endoplasmic reticulum and modulated the repair pathway. Cell survival after FLASH-RT was no different from CONV-RT. For Pan02 cells, FLASH-RT may create a more immunostimulatory environment with low levels of suppressive proteins (PD-L1) and high levels of immunostimulatory proteins (CRT and MHC-I). The main mechanism underlying the difference between the two cell lines remains unclear [177]. For tumors located at greater depth, heavy ion FLASH-RT experiments were performed. FLASH-RT with raster-scanning helium ion beams at a dose rate of 141 Gy/s at a dose of 10 Gy to the head of healthy C57BL/6 mice revealed a significant reduction in acute brain tissue damage (as measured by γH2AX), structural preservation of neurovascular endothelium (CD31+), and a decrease in the activation of neuroinflammatory signals (detected by quantifying CD68+ and microglia/macrophage-inducible nitric oxide synthetase expression) compared to the CONV regimen (0.2 Gy/s) [24]. In addition to these results, the irradiation of tumors with 12.5 Gy of helium ions (A549 carcinoma cells inoculated into the mouse leg) revealed a significant reduction in tumor volume at day 14 post-irradiation for both FLASH-RT (250 Gy/s) and CONV-RT (0.2 Gy/s). Mice survival was higher for the FLASH-RT group (43 days post-irradiation period) than for those irradiated in the CONV mode (24 days) [24]. In experiments irradiating mouse hind limbs (one limb bearing a tumor, the other without) with carbon ions in SOBP (240 MeV/nucleon) using FLASH-RT or CONV-RT at a dose of 20 Gy, muscle damage (fibrosis and collagen deposition) in the healthy limb and skin toxicity were less with FLASH-RT than with CONV-RT. However, no differences in the overall generalized immune response were observed between these modalities. Both irradiation modes resulted in an increase in cytotoxic T cells (CD8+) in the tumor 28 days after irradiation. Furthermore, irradiation in both modes restored the gut microbiome to a healthy state. Equivalent tumor control was observed for both modes. It should be noted that in these experiments, the non-irradiated parts of the mouse body were shielded with brass absorbers and the mice were anesthetized with isoflurane, which may have reduced the abscopal effect [9].

### 3.5. Medical Physics Variations: Dose Limit, Hypofractionation, Impulse Structure, and Reirradiation

As can be seen from the analysis of the literature data of recent years, a single irradiation with FLASH-RT, which resulted in faster recovery of normal tissues and an increase in the lifespan of animals compared to CONV-RT while also maintaining similar tumor growth control as CONV-RT, was mainly in the range of 10–25 Gy with electron irradiation [4,13,14,20,22,39,167], protons [50,175,176], helium ions [24], and carbon ions [9]. At single doses of 30 Gy and higher, in some studies, radiation responses to electron irradiation according to skin toxicity and survival criteria between FLASH-RT and CONV-RT did not differ significantly [4,13]. A comparison of acute skin toxicity (hair loss, moist scaling, and toe separation) in intact, unanesthetized mice locally irradiated (hind leg) with protons (ProBeam, Varian, Siemens Healthineers, Palo Alto, CA, USA) in FLASH (60 Gy/s) or CONV (0.34 Gy/s) modes at high doses (single doses of 19.9 to 49.7 Gy for CONV and 30.4 to 65.9 Gy for FLASH) showed a sparing effect of FLASH-RT compared to a single CONV-RT with an average sparing factor (SF) of 1.40. The development of fibrosis at late stages after irradiation (30 weeks) also showed a sparing FLASH effect on normal tissue with SF = 1.18 [51]. When treating dogs with superficial tumors with single-electron irradiation in the range of 15–35 Gy in the FLASH mode, a dose of 35 Gy was found to be toxic. Moreover, the observation was carried out for 12 months post-irradiation period. The authors believe that a dose of 30 Gy is apparently the maximum safe single dose for FLASH-RT [32]. It should be noted that this dose was used for proton irradiation of a mouse limb. When irradiating a rat’s head with an electron beam, a dose of 30 Gy was too high and resulted in decreased survival compared to 20 and 25 Gy [4]. Comparative studies of different IR sources showed similar FLASH-sparing. The acute response to electron irradiation (16 MeV) was similar to previous reports with proton irradiation; the mouse leg was irradiated with single doses of 19.4–57.6 Gy in either CONV (0.162 Gy/s) or FLASH (233 Gy/s) modes. The development of acute skin toxicity was similar between the CONV and FLASH modes at biologically equivalent doses in terms of SF = 1.45–1.54. A lesser sparing effect was observed for the fibrosis development criterion, SF = 1.15 [178]. Dose hypofractionation can modify the magnitude of the protective FLASH effect. Total abdominal irradiation of C57BL/6J mice with an electron beam (IntraOp Medical, Sunnyvale, CA, USA, 9-MeV) at comparable doses of 11–14 Gy either as a single fraction or with different combinations of dose fractions and dose rates up to ultra-high dose rate beam conditions, and a comparison of proton irradiation at plateau and in SOBP with electron irradiation revealed a strong influence of beam parameter settings on the magnitude of the FLASH effect [18,56]. For scanning with a proton pencil beam (p-PBS), FLASH-RT showed reduced skin toxicity in mice and fibrosis when delivered as a single continuous high-dose fraction of 30, 35, and 40 Gy. At 30 and 35 Gy, a 2 min interruption of 2 × 15 Gy or 2 × 17.5 Gy fractions reduced the tissue-sparing FLASH effect, which remained statistically significant. However, two interruptions of 3 × 10 Gy or 3 × 11.6 Gy fractions completely eliminated the tissue-sparing FLASH effect. This delivery method was used because clinical p-PBS treatment typically requires multiple beams to achieve good conformality, and these beams are separated by minutes to allow for patient repositioning. The right hind legs of 10-week-old female C57Bl/6j mice were irradiated using a Varian ProBeam proton beam scanning gantry system at conventional (1 Gy/s) or FLASH (100 Gy/s) mean dose rates [49]. Similar results—decreasing tissue sparing proportional to the number of fractions into which the total dose was split—have been obtained by other researchers even for a single 2 min pause [163]. The next study of threshold duration of pause between hypofractions revealed that 15 s and more are enough to lose FLASH-sparing whereas 5 s and less are sufficient to achieve as much FLASH-sparing as a single pulse does [101].

The loss of FLASH-mode driven tissue sparing after the subfractionation of a dose raises questions to persisting FLASH-sparing for pencil beam applications. Experimental data postulated that pencil beam proton irradiation in FLASH mode provided similar tumor control to CONV-RT, but reduced normal tissue damage, assessed as acute skin damage and radiation-induced fibrosis [26,179]. Quantitative assessment of the sparing effects of FLASH irradiation on acute (hair loss, moist desquamation, and toe separation) and late (development of fibrosis—leg extension) skin toxicity following leg irradiation in healthy, unanesthetized female CDF1 mice (single fraction irradiation with doses ranging from 19.9 to 49.7 Gy for CONV and from 30.4 to 65.9 Gy for FLASH) was made using a proton pencil beam (ProBeam, Varian, Siemens Healthineers, CA, USA) in the SOBP region. Comparison of acute skin toxicity after irradiation showed a sparing effect of FLASH-RT compared to CONV-RT with a mean SF = 1.40 (1.35–1.46). Fibrous development also showed a sparing effect on normal tissue with SF = 1.18 (1.17–1.18) [51].

The relationship between the average dose rate and the dose per pulse was examined in detail for gastrointestinal toxicity induced by electron irradiation of the abdominal region of mice. The study showed that both a high dose per pulse and an ultra-high mean dose rate were independently sufficient to achieve a gentle FLASH effect (reduced gastrointestinal toxicity), while an isoeffective tumor response was maintained across all irradiation modes including CONV or low-pulse dose and low-mean dose rate [18].

In studies of chronic toxicity induced by repeated CONV-RT or FLASH-RT with protons, FLASH-RT was found to induce less intestinal fibrosis and collagen deposition in the intestine, skin, and bone of mice compared to CONV-RT, which was accompanied by significantly higher survival, as well as less edema, lower levels of fibrous connective tissue in the skin, and fewer bone fractures. A single intestinal irradiation was administered at a dose of 12 Gy for CONV-RT, followed by a second dose of 12 Gy in either the FLASH or CONV modes. In addition, a hypofractionated schedule (3 × 6.4 Gy) was used for repeat irradiation every 48 h for FLASH-RT or CONV-RT. To evaluate the effects (in terms of leg skin/bone sparing) of re-irradiation with 15 Gy, previously irradiated CONV-RT mice were re-irradiated with hypofractionated FLASH-RT or CONV-RT (3 × 11 Gy). These studies provide the first evidence of the sparing effect of FLASH under hypofractionated re-irradiation [52].

### 3.6. Combining FLASH-RT with Other Treatments

Although FLASH-RT has not yet become a common medical technology, options for combining it with other types of anticancer treatments are already being proposed. The dosimetric feasibility and quality of planning for combined exposure to ultra-high dose rates of electrons and conventional dose rates of photons, as well as the combination of FLASH-RT with other treatment options, including spatially fractionated radiotherapy, immunotherapy, and the use of FLASH-RT in the setting of re-irradiation, are being studied [180,181]. Some recommendations are offered to promote the progress of FLASH-RT in nanomedicine approaches [182].

## 4. Conclusions

The dominant factors that influence the magnitude of the FLASH effect are still being discussed, but it is clear that the following of them are important: the choice of the type of ionizing radiation and the method of dose delivery, including a single dose delivery and minimization of the number of fractions for the total dose, high-quality and adequate dosimetry, assessment of the volume of irradiated blood as a factor in the off-target effect, the method of anesthesia, the level of tissue oxygenation, consideration of sex and individual differences in individuals, and the use of radiosensitizers that are non-toxic to normal tissue. Other factors likely exist. The literature data also suggests that FLASH-RT induces effects at the cellular and tissue level that are mediated by the modulation of signaling pathways [9,27,50,172,176,183], which is likely to be actively studied in the near future.

In an irradiated cell or organism, several mechanisms of radiation exposure are simultaneously realized at the physicochemical, molecular biological, biochemical, cellular, and organismal levels. Based on this, and the fact that different researchers have used various types of accelerators and radiation, different dose rates, delivery methods, and dosimetric monitoring, it is clear that more extensive research into the FLASH effect is needed in various fields.

Given the observed preservation of normal tissue, clinically relevant studies of the FLASH effect should be conducted not only in tumor models but also in normal tissue irradiated in the SOBP region. This is important due to the imperfect conformability of SOBP even when using individual collimators, as well as for dose fractionation purposes and for studying the effects of secondary radiation generated during the generation of ultra-high dose rates of ionizing radiation [184,185].

It should be noted that each tumor has individual characteristics, the impact of which on the radiation response can be difficult to assess. To identify the characteristics of the radiation response, various cell cultures with varying levels of radiation resistance and oxygenation are used, as well as animal models—anesthetized or not, of different sexes, with different metabolic rates, hematopoiesis types, immune status, and lifespan. When studying blood, it is necessary to consider the characteristics of the hematopoietic system of living organisms. For example, mice have lymphoid hematopoiesis and a predominance of lymphocytes among blood leukocytes, unlike humans (myeloid hematopoiesis, a predominance of neutrophils among leukocytes). Lymphocytes are known to be the most radiosensitive of all blood leukocytes. The short lifespan of mice and rats and the occurrence of spontaneous tumors near the end of life in intact animals preclude assessing the long-term effects of radiation exposure in these models. In this regard, the use of larger domestic animals with spontaneous tumors undergoing treatment in veterinary clinics becomes important, which may be suitable for identifying the effects of ultra-high dose rate irradiation [32]. The use of biomaterial obtained from tumor-bearing patients is also advisable.

A comparative study of pulsing showed that the radioprotective FLASH effect in terms of reducing post-radiation inflammation and improving intestinal crypt regeneration can be achieved at a high instantaneous dose per pulse (≥1.02 × 10^5^ Gy/s) not only at a high average dose rate (>40 Gy/s), but also at a low average dose rate (0.4 Gy/s) [41]. Thus, inaccurate dosimetry and underestimation of the instantaneous pulse power in some studies may lead to a false-negative absence of the radioprotective FLASH effect. It should be noted that despite the large number of studies confirming the presence of the FLASH effect when exposed to radiation with different LETs, there are also other results that found only little or no difference in tumor growth delay or IR-induced toxicity with proton FLASH therapy compared with proton irradiation at a conventional dose rate, including when comparing proton irradiation with electron irradiation at different dose rates [9,18,53,56,84]. All of this requires further research into the mechanisms and methods for increasing the effectiveness of the FLASH mode in radiotherapy.

The lack of data on the effects of FLASH radiation on humans (except for isolated cases) is understandable. Currently, proton therapy with a traditional dose rate is increasingly used clinically due to the spatial distribution of the dose within the patient’s body. However, a common problem in clinical proton therapy is the uncertainty of the calculated proton range, for example, when calculating the settings of a treatment device for a patient. The uncertainty of the range may depend on knowledge of the proton beam’s energy distribution and the properties of all absorbing materials in the beam path on the accuracy of the measuring equipment, and, in some cases, on the skill of the experimenter [186]. With the variety of irradiation setups used in preclinical studies on animal models, questions related to the variability of the pulse structure also remain open.

Some practical recommendations are as follows:

• Reporting standards must be followed [79].
• To understand the resulting radiation effects, beam parameters must be taken into account (where possible), including beam structure and instantaneous dose rate.
• An assessment of the interaction of radiation with laboratory equipment materials located in the beam path is desirable. The use of protective shields is possible.
• Reliable in-beam/in vivo measurements of O_2_ and ROS are a priority.
• Anesthesia and animal sex controls are necessary in preclinical trials, as well as pO_2_ monitoring.
• To ensure the FLASH effect is as gentle as possible on normal tissue, the number of hypofractions must be reduced.
• It is also necessary to consider and match the target boundaries with the SOBP boundaries for heavy ions.

## Figures and Tables

**Table 1:** FLASH mode control tumor equal to CONV (superiority in tumor growth control).

Species	Graft	Tumor Control	IR Type	Bragg	Reference
*Rattus rattus*	NS1	comparable	electrons	NA	[3]
*Rattus rattus*	NS1	comparable	electrons	NA	[4]
*Rattus rattus*	glioma	comparable	protons	-	[5]
*Rattus rattus*	RG2	comparable	protons	-	[6]
*Mus musculus*	A549	comparable	na/nr	NA/NR	[7]
*Mus musculus*	LM8	comparable	^12^C-ions	-	[8]
*Mus musculus*	LM8	-	^12^C-ions	+	[9]
*Mus musculus*	MDA-MB 231	NA	electrons	NA	[10]
*Mus musculus*	A549, Calu6	comparable	electrons	NA	[11]
*Mus musculus*	GL261	comparable	electrons	NA	[12]
*Mus musculus*	GL261, B16F10	comparable	electrons	NA	[13]
*Mus musculus*	H3.3K27M	comparable	electrons	NA	[14]
*Mus musculus*	H454	fewer lung metastases	electrons	NA	[15]
*Mus musculus*	ID8	comparable	electrons	NA	[16]
*Mus musculus*	ID8, UPK10	comparable	electrons	NA	[17]
*Mus musculus*	melanoma Mus m.	-	electrons	NA	[18]
*Mus musculus*	Py8119, Py230	comparable	electrons	NA	[19]
*Mus musculus*	SV2-OVA, GL261, H454, U-87, mEERL95	comparable	electrons	NA	[20]
*Mus musculus*	U-87	NA	electrons	NA	[21]
*Mus musculus*	U-87, SV2, mEERL95	superiority with trametinib	electrons	NA	[22]
*Mus musculus*	T-LBAL	-	electrons	NA	[23]
*Mus musculus*	A549	comparable	helium ions	+	[24]
*Mus musculus*	MH641905	comparable	protons	-	[25]
*Mus musculus*	C3H	-	protons	-	[26]
*Mus musculus*	GL261	-	protons	NR	[27]
*Mus musculus*	LSL-KrasG12D/wt;p53FL/FL	superior in 2 out of 3 cases	protons	-	[28]
*Mus musculus*	MOC2-luc	comparable	protons	-	[29]
*Mus musculus*	EMT6	comparable	X-rays	NA	[30]

Table legend: +—fractionation, NA/-—not applicable, NR—not registered, NA/NR—unavailable.

**Table 2:** Diversity of living models and irradiation equipment in FLASH mode experiments.

Species	FLASH-Sparing	Doses (Gy) for Sparing	IR Type	Equipment	Particle Energy	Reference
*Homo sapiens*	Yes	NA	electrons	Oriatron eRT6	5.6 MeV	[31]
*Canis lupus familiaris*	NA	NA	electrons	Elekta AB	10 MeV	[32]
*Canis lupus familiaris*	NA	NA	electrons	Elekta AB	10 MeV	[33]
*Felis catus*	Yes	NA	electrons	eRT6/Oriatron	6, 9, or 12 MeV	[34]
*Felis catus*	NA	NA	electrons	linacs of type Kinetron and Oriatron 6e,	4.5 MeV and 6 MeV	[35]
*Sus scrofa*	NA	NA	electrons	eRT6/Oriatron	6, 9, or 12 MeV	[34]
*Sus scrofa*	Yes	28–34	electrons	linacs of type Kinetron and Oriatron 6e,	4.5 MeV and 6 MeV	[35]
*Rattus rattus*	Yes	-	electrons	Elekta AB	10 MeV	[4]
*Rattus rattus*	No	-	electrons	Elekta AB, Sweden	10 MeV	[3]
*Rattus rattus*	Yes (oxygen free)	25	protons	nozzle-equipped gantry Orsay proton therapy center (ICPO)	226 MeV	[6]
*Rattus rattus*	Yes	-	protons	nozzle-equipped gantry Orsay proton therapy center (ICPO)	NA/NR	[5]
*Mus musculus*	Yes (fibrosis)	NA/NR	^12^C-ions	NA/NR	NA/NR	[9]
*Mus musculus*	Yes	NA/NR	^12^C-ions	NA/NR	240 MeV/nuclon	[8]
*Mus musculus*	Yes	14, 16	electrons	Varian Medical Systems	16 MeV	[16]
*Mus musculus*	Yes	4	electrons	eRT6/Oriatron	6 MeV	[23]
*Mus musculus*	Yes	10, 2 × 7, 3 × 10	electrons	LINAC/type Oriatron 6e	6 MeV	[15]
*Mus musculus*	Yes	14	electrons	Varian Medical Systems	16 MeV	[17]
*Mus musculus*	Yes	25	electrons	Megavoltage electron beams	9–10 MeV	[13]
*Mus musculus*	Yes	7, 10, 12.5	electrons	LINAC	6 MeV	[36]
*Mus musculus*	Yes	2 × 10	electrons	Oriatron 6e	6 MeV	[37]
*Mus musculus*	Yes	10	electrons	Oriatron 6e	6 MeV	[19]
*Mus musculus*	Yes	10	electrons	Chengdu THz Free Electron Laser	6 MeV	[12]
*Mus musculus*	Yes	12, 14	electrons	Varian Clinac 2100C	9 MeV	[18]
*Mus musculus*	Yes	27	electrons	LINAC	9 MeV	[38]
*Mus musculus*	Yes	10	electrons	Mobetron	9 MeV	[39]
*Mus musculus*	Yes	18	electrons	UHDR vertical test platform	6 Mev	[40]
*Mus musculus*	Yes	8, 10	electrons	An electron linear accelerator	6 MeV	[11]
*Mus musculus*	Yes	10	electrons	Elekta Infinity linac	4.7, 5.3 MeV	[41]
*Mus musculus*	Yes	20	electrons	Oriatron 6e	6 MeV	[22]
*Mus musculus*	No	-	electrons	Oriatron 6e	5.5 MeV	[42]
*Mus musculus*	No	-	electrons	Oriatron eRT6	NR	[43]
*Mus musculus*	-	-	electrons	Varian Clinac 2100 C/D	10 MeV	[10]
*Mus musculus*	-	No	electrons	Oriatron eRT6	5.5 MeV	[20]
*Mus musculus*	-	-	electrons	IBA Proteus Plus Cyclotron	230 MeV	[14]
*Mus musculus*	-	10	electrons	LINAC	6 MeV	[44]
*Mus musculus*	-	40	electrons	LINAC	6 MeV	[21]
*Mus musculus*	-	-	electrons	LINAC	6 MeV	[45]
*Mus musculus*	Yes	10	helium ions	NA/NR	NA/NR	[24]
*Mus musculus*	Yes	-	NA/NR	NA/NR	NA/NR	[7]
*Mus musculus*	No	5.1–5.4 (TBI)	photons	IMBL	95 keV–124 keV	[46]
*Mus musculus*	Yes	30, 45	protons	BA Proteus Plus C230 Cyclotron,	230 MeV	[28]
*Mus musculus*	Yes	15	protons	IBA Proteus Plus	230 MeV	[25]
*Mus musculus*	Yes	40–60	protons	ProBeam, Varian Medical Systems,	244–250 MeV	[26]
*Mus musculus*	Yes	25	protons	Ion Beam Applications SA	230 MeV	[47]
*Mus musculus*	Yes	10.5–16.3	protons	S250i; Mevion Medical Systems	230 MeV	[48]
*Mus musculus*	Yes	30, 35, 40, 2 × 15, 2 × 17.5	protons	Mobetron linear accelerator	9 MeV	[49]
*Mus musculus*	Yes	14	protons	SIS18 synchrotron	240 MeV	[50]
*Mus musculus*	Yes	14, 16, 18, 3 × 8	protons	IBA Proteus Plus Cyclotron	230 MeV	[29]
*Mus musculus*	Yes	30.4–49.7	protons	ProBeam, Varian	244–250 MeV	[51]
*Mus musculus*	Yes	10	protons	LINAC	15.73 MeV, 16.60 MeV	[27]
*Mus musculus*	Yes	12 + 12, 12 + 3 × 6.4, 15 + 3 × 11	protons	IBA Proteus Plus C230 Cyclotron	230 MeV	[52]
*Mus musculus*	Vice versa	14	protons	Mobetron electron linear accelerator	9 MeV	[53]
*Mus musculus*	No	15.1, 16.2, 17, 18	protons	Ion Beam Applications SA	228 MeV	[54]
*Mus musculus*	Yes	16	protons	Ion Beam Applications SA	228.9 MeV.	[55]
*Mus musculus*	Vice versa	12–14	protons and electrons	Hitachi synchrotron	87 MeV	[56]
*Mus musculus*	Yes	13	X-rays	Rad Source; RS-2000 Pro, PARTER	-	[57]
*Mus musculus*	Yes	12 (intestine), 30 (lung)	X-rays	PARTER platform at the CTFEL	6–8 MeV	[30]
*Coturnix coturnix japonica*	Yes	4.5, 8	protons	linear proton accelerator of INR RAS	NR	[58]
*Gallus gallus*	Yes	10–14	protons	PROBEAT III	139 MeV	[59]
*Danio rerio*	Yes	-	electrons	NA/NR	NA/NR	[60]
*Danio rerio*	Yes	26–27	electrons	ELBE	NA/NR	[61]
*Danio rerio*	Yes	-	helium ions	NA/NR	NA/NR	[62]
*Danio rerio*	Yes	22.5	protons	A horizontal fixed-beam beamline in the UPTD	224 MeV	[63]
*Danio rerio*	Yes	30	protons		NA/NR	[64]
*Danio rerio*	Yes	15–50	protons and electrons	UPTD and ELBE	225 MeV (protons), 30 Mev (electrons)	[65]
*Drosophila melanogaster*	Yes	22	X-rays	X-ray tube	-	[66]
*Caenorhabditis elegans*	Yes	10, 20	Protons, electrons and X-rays	ALTAÏS UHDR Mobetron, and X-Rad 225XL	4 Mev (protons) 9 MeV (electrons)	[67]

Table legend: +—fractionation, ×—hypofractionation, TBI—total body irradiation, NA/-—not applicable, NR—not registered, NA/NR—unavailable.

**Table 3:** Radiolysis products and oxygen levels during irradiation of water or solutions with different dose rates.

Radiation Source	Radiation Type	LET	Dose Rate	Target	Radiolysis Products/Oxygen	Radiolysis Product Concentrations	Link
GE PETtrace Cyclotron University of Wisconsin, Madison, WI, USA	Proton 9.7 MeV	Starts with—12.8 eV/nm	10–10^4^ Gy/s 2.23 × 10^12^–7.50 × 10^13^ eV/impulse	Water in a closed container. Water in a flow-through container.	H_2_, H_2_O_2_, O_2_ e_aq_^−^, H, HO_2_, O_2,_ HO	The calculated steady-state concentrations of all radiolysis products in a closed container increase with increasing dose rate at 1 μA. The highest concentrations at 1000 s were observed for H_2_ and H_2_O_2_ (approximately 10^−4^ M); during the irradiation process the water became acidified to pH 5.84. Levels of radiolytic products were monitored in a flow-through tank for 100 ms, with the highest level observed for H_2_ and H_2_O_2_ at 20 μA.	[134]
10 MeV FN Tandem Van de Graaff, Davis, CA, USA	Proton 2 MeV and 10 MeV Helium ion, 5 MeV	34.8 eV/nm and 13.8 eV/nm 156 eV/nm	0.20 Gy/s and 1 Gy/s 0.25 Gy/s	Water in a closed container.	e^−^_aq_, H, OH, H_2_, H_2_O_2_, HO_2_	H_2_O_2_ formation increases with increasing LET and proton dose. H_2_O_2_ concentration is approximately four times higher at 2 MeV compared to 10 MeV. The concentration of H_2_O_2_ is approximately three times higher when irradiated with helium ions (5 MeV) compared to protons (2 MeV).	[137]
PANTAK 320S (Shimadzu), Kyoto, Japan HIMAC, (National Institute of Radiological Sciences), Chiba, Japan	X-ray (200 kV, 20 mA) 80 keV Carbon ion 290 MeV/nuclon	13, 20, 40, 60, 80 or >100 keV/μm	3.04–3.15 Gy/min -	Water (milli-Q) in a closed container.	H_2_O_2_	Under hypoxic conditions (<0.5% O_2_), H_2_O_2_ release is minimal at a dose of 64 Gy. Higher LET carbon ions can create more H_2_O_2_ in an O_2_-independent manner than lower LET radiation and/or X-ray photons at a dose of 64 Gy.	[132]
ARRONAX cyclotron, Saint-Herblain, France	Proton		0.2 Gy/s and 40 Gy/s–60 kGy/s	Water in a closed container.	H_2_O_2_	A significant decrease in H_2_O_2_ production was observed from 0.2 to 1.5 kGy/s with a sharper decrease below 100 kGy/s.	[131]
Marburg Ion-Beam Therapy Center, Germany A 9 MeV electron source generated by a Mobetron unit (IntraOpMedical, Sunnyvale, CA, USA) MultiRad 225 (Precision, Minneapolis, MN, USA)	Carbon ion 430.1 MeV/nuclon Electron X-ray (200 kV, 17.8 mA)		>40 Gy/s 600 Gy/s and 0.62 Gy/s 0.1 and 10 Gy/s	Water (milli-Q) in a closed container: real hypoxic—1% O_2_, hypoxic—1% O_2_ + 5% CO_2_ and normoxic—21% O_2_.	H_2_O_2_	For water with varying O_2_ and CO_2_ concentrations, high dose rate irradiation always reduces H_2_O_2_ production compared to conventional dose rates, regardless of LET. O_2_ and CO_2_ can increase H_2_O_2_ production, and e^−^_aq_ scavengers (N_2_O and NaNO_3_) can reduce the difference in H_2_O_2_ production between high and conventional dose rates.	[133]
AVF-930 cyclotron National Institutes for Quantum and Radiological Science and Technology (QST), the National Institute of Radiological Sciences (NIRS), Chiba, Japan	Proton 27.5 MeV Proton 27.5 and 55 MeV Carbon ion 140 MeV/nuclon Proton 230 MeV		0.05–160 Gy/s ≤100 Gy/s >40 Gy/s	Solutions of coumarin-3-carboxylic acid and ferrocyanide.	e^−^_aq_ and •OH	The yields of e^−^_aq_ and •OH decrease with increasing dose rate. The minimum value of the radical yield in the Bragg peak is considered to be the result of radical recombination caused by high LET values.	[118,135,136]
UHDR (LINAC) Mobetron, Fremont, CA, USA	Electron 9 MeV		Instantaneous dose rate 0.18–0.33 MGy/s	Water.	e^−^_aq_	The instantaneous dose rate directly influenced the yield of hydrated electrons during water radiolysis under flash irradiation.	[138]
Varian Trilogy, Palo Alto, CA, USA	Electron 10 MeV		Mean dose rate: 0.14–1500 Gy/s	An air-equilibrated aqueous solution of 4% bovine serum albumin in the presence of Oxyphor, PdG4, or Amplex Red^®^ , in a closed container.	H_2_O_2_, O_2_	As the mean dose rate increased, a significant decrease in the rates of oxygen consumption and hydrogen peroxide formation was observed; oxygen consumption was dependent on the initial pO_2_ for CONV and FLASH-irradiation, with oxygen consumption decreasing as the initial pO_2_ decreased.	[102]
Faxitron MultiRad225, Faxitron Bioptics, LLC Heidelberg Ion beam Therapy facility HIT, Germany OncoRay, Dresden, Germany	X-ray (225 kV) Carbon ion 400 MeV/nuclon and 150 MeV/nuclon Proton 224 MeV/nuclon	~1.7 keV/μm 10.89 and 19.47 keV/μm 0.42 keV/μm	0.03–52 Gy/s 0.06–2.4 and 0.04–1.8 Gy/s 0.03–340 Gy/s	Water (milli-Q) in a closed container.	O_2_	Complete oxygen depletion for photons, protons, and carbon ions was not observed in FLASH-irradiated water samples at a dose of 10 Gy. At higher dose rates, less oxygen was consumed than at standard dose rates.	[95]
Varian Clinac 2100 C/D, Palo Alto, CA, USA	Electron 10 MeV		0.1 Gy/s or 270–300 Gy/s	0.5% albumin solution in PBS with Oxyphor 2P. Mice injected subcutaneously with MDA-MB 231 cells in presence of Oxyphor 2P.	O_2_	Oxygen depletion in flash-irradiated albumin solutions or tumor/normal tissues is dose-dependent and independent of dose rate within the range of 50–300 Gy/s for the solution and 90–270 Gy/s for tissues. Oxygen depletion is greater in normal tissues than in tumor tissues. Changes in tissue pO_2_ range from 1 to 3 mmHg at doses of 20 Gy and 270 Gy/s, which is insufficient to induce volumetric hypoxia in normally oxygenated tissues.	[10]
Mobetron (IntraOp); HIT (Heidelberg Center for Ion Beam Therapy), Germany	Electron 9 MeV, proton 146.56 MeV, helium ion 145.74 MeV/nuclon, carbon ion 275.98 MeV/nuclon, oxygen ion 325.98 MeV/nuclon	1, 5.4, 14.4, 65 and 100.3 keV/μm	0.3–0.4 Gy/s and 100 Gy/s.	Air-equilibrated 5% bovine serum albumin solution in DPBS in a closed container.	O_2_	In samples irradiated at a dose of 15 Gy, the oxygen consumption rate decreases with increasing LET from 0.351 mmHg/Gy for low-LET electrons to 0.1796 mmHg/Gy for high-LET oxygen ions at the conventional dose rate and from 0.317 to 0.1556 mmHg/Gy for the FLASH mode, respectively. The higher consumption rate for CONV irradiation compared to FLASH irradiation was maintained for all particle types.	[96]
ProBeam, Varian, Siemens Healthineers, Malvern, PA, USA	Proton, pencil beam, 244 MeV		Field dose rate—1.2–2.7 Gy/s, maximum instantaneous dose rate ~40 Gy/s	Saline solution containing Oxyphor PtG4 probe.	O_2_	The rate of decrease in pO_2_ is directly related to the local instantaneous dose rate.	[98]
IBA Proteus Plus, Louvain-la-Neuve, Belgium	Proton, 230 MeV		0.5–100 Gy/s	Oxyphor PtG4 in HEPES containing glycerol, glucose, and glutathione; female C57BL/6 mice with fibrosarcoma cells injected subcutaneously into the thigh *LSL-Kras^G12D/wt^*; *p53^FL/F^* GEMM.	O_2_	FLASH irradiation of a solution simulating the intracellular environment depleted oxygen by approximately 25% per Gy compared to CONV irradiation. In vivo, oxygen depletion during irradiation was compensated for by replenishment from the vascular system; tissue pO_2_ remained virtually unchanged at the CONV dose rate. FLASH irradiation was accompanied by a stepwise decrease in pO_2_, followed by a rebound to baseline after approximately 8 s. In solutions simulating the cellular environment, oxygen depletion G-values were significantly higher than those observed in vivo.	[97]

**Table 4:** Reduced tissue-specific toxicity in FLASH experiments.

Species	FLASH Sparing	Doses (Gy) for FLASH-Sparing	IR Type	Reference
Survival				
*Mus musculus*	Yes	14, 16	electrons	[16]
*Mus musculus*	Yes	10	electrons	[41]
*Mus musculus*	Yes	10.5–16.3	protons	[48]
*Mus musculus*	Yes	14	protons	[50]
*Mus musculus*	Yes	14, 16, 18, 3 × 8	protons	[29]
*Mus musculus*	Yes	12 + 12, 12 + 3 × 6.4, 15 + 3 × 11	protons	[52]
*Mus musculus*	Vice versa	14	protons	[53]
*Mus musculus*	Yes	16	protons	[55]
*Mus musculus*	Vice versa	12–14	protons and electrons	[56]
*Mus musculus*	Yes	12 (intestine), 30 (lung)	X-rays	[30]
Weight Loss				
*Mus musculus*	Yes	14, 16	electrons	[16]
*Mus musculus*	No	5.1–5.4 (TBI)	photons	[46]
*Mus musculus*	Yes	14	protons	[50]
*Mus musculus*	Yes	16	protons	[55]
Fibrosis				
*Mus musculus*	Yes	-	^12^C-ions	[9]
*Mus musculus*	Yes	15	protons	[25]
*Mus musculus*	Yes	12 + 12, 12 + 3 × 6.4, 15 + 3 × 11	protons	[52]
*Mus musculus*	Yes	40–60	protons	[26]
*Mus musculus*	Yes	30.4–49.7	protons	[51]
*Mus musculus*	Yes	30, 35, 40, 2 × 15, 2 × 17.5	protons	[49]
*Mus musculus*	Yes	14, 16, 18, 3 × 8	protons	[29]
*Mus musculus*	Yes	13	X-rays	[57]
Skin Toxicity				
*Canis lupus familiaris*	NA	NA	electrons	[32]
*Canis lupus familiaris*	NA	NA	electrons	[33]
*Felis catus*	Yes	NA	electrons	[34]
*Homo sapiens*	Yes	-	electrons	[31]
*Mus musculus*	Yes	20	^12^C-ions	[9]
*Mus musculus*	Yes	25	electrons	[13]
*Mus musculus*	Yes	27	electrons	[38]
*Mus musculus*	Yes	30, 45	protons	[28]
*Mus musculus*	Yes	40–60	protons	[26]
*Mus musculus*	Yes	25	protons	[47]
*Mus musculus*	Yes	30, 35, 40, 2 × 15, 2 × 17.5	protons	[49]
*Mus musculus*	Yes	30.4–49.7	protons	[51]
*Mus musculus*	Yes	12 + 12, 12 + 3 × 6.4, 15 + 3 × 11	protons	[52]
*Mus musculus*	Yes	12 (intestine), 30 (lung)	X-rays	[30]
*Rattus rattus*	Yes	20	electrons	[4]
*Rattus rattus*	No	-	electrons	[3]
*Rattus rattus*	NA/NR	NA/NR	protons	[6]
*Sus scrofa*	Yes	28–34	electrons	[35]
*Sus scrofa*	NR	NR	electrons	[34]
Intestinal Toxicity				
*Mus musculus*	Yes	14	electrons	[17]
*Mus musculus*	Yes	7, 10, 12.5	electrons	[36]
*Mus musculus*	Yes	10	electrons	[19]
*Mus musculus*	Yes	12, 14	electrons	[18]
*Mus musculus*	Yes	8, 10	electrons	[11]
*Mus musculus*	Yes	10	electrons	[41]
*Mus musculus*	Yes	-	NA\NR	[7]
*Mus musculus*	Yes	15	protons	[25]
*Mus musculus*	Yes	14	protons	[50]
*Mus musculus*	Vice versa	14	protons	[53]
*Mus musculus*	No	15.1, 16.2, 17, 18	protons	[54]
*Mus musculus*	Vice versa	12–14	protons and electrons	[56]
Cognition and neurogenesis				
*Mus musculus*	No	-	electrons	[43]
*Mus musculus*	Yes	10, 2 × 7, 3 × 10	electrons	[15]
*Mus musculus*	Yes	2 × 10	electrons	[37]
*Mus musculus*	Yes	10	electrons	[12]
*Mus musculus*	Yes	10	electrons	[39]
*Mus musculus*	Yes	10	electrons	[39]
*Mus musculus*	Yes	10	electrons	[39]
*Rattus rattus*	Yes	-	protons	[5]
Inflammation				
*Mus musculus*	Yes	10	electrons	[41]
*Mus musculus*	Yes	2 × 10	electrons	[37]
*Mus musculus*	Yes	10	electrons	[39]
*Mus musculus*	Yes	10	electrons	[39]
*Mus musculus*	Yes	10	helium ions	[24]
*Mus musculus*	Yes	30, 45	protons	[28]
*Mus musculus*	Yes	12 + 12, 12 + 3 × 6.4, 15 + 3 × 11	protons	[52]
Blood composition and hematopoesis				
*Mus musculus*	Yes	4	electrons	[23]
*Mus musculus*	Yes	10	electrons	[41]
*Mus musculus*	Yes	30, 45	protons	[28]
*Mus musculus*	Yes	12 + 12, 12 + 3 × 6.4, 15 + 3 × 11	protons	[52]
*Mus musculus*	Yes	10	protons	[27]
*Mus musculus*	No	15.1, 16.2, 17, 18	protons	[54]
Lung Toxicity				
*Mus musculus*	Yes	-	NA/NR	[7]
*Mus musculus*	Yes	18	electrons	[40]
*Mus musculus*	Yes	12 (intestine), 30 (lung)	X-rays	[30]
*Homo sapiens*	No	-	protons	[147]
Osteotoxicity and muscle degradation				
*Felis catus*	Yes	NA	electrons	[34]
*Mus musculus*	Yes	NA/NR	^12^C-ions	[8]
*Mus musculus*	Yes	12 + 12, 12 + 3 × 6.4, 15 + 3 × 11	protons	[52]
*Mus musculus*	Yes	30, 45	protons	[28]

Table legend: +—fractionation, ×—hypofractionation, TBI—total body irradiation, NA/-—not applicable, NR—nor registered, NA/NR—unavailable.

## Data Availability

No new data were created or analyzed in this study. Data sharing is not applicable to this article.

## References

[1] Xiang M., Chang D.T., Pollom E.L. (2020). Second Cancer Risk after Primary Cancer Treatment with Three-dimensional Conformal, Intensity-modulated, or Proton Beam Radiation Therapy. Cancer.

[2] Favaudon V., Caplier L., Monceau V., Pouzoulet F., Sayarath M., Fouillade C., Poupon M.-F., Brito I., Hupé P., Bourhis J. (2014). Ultrahigh Dose-Rate FLASH Irradiation Increases the Differential Response between Normal and Tumor Tissue in Mice. Sci. Transl. Med..

[3] Konradsson E., Liljedahl E., Gustafsson E., Adrian G., Beyer S., Ilaahi S.E., Petersson K., Ceberg C., Nittby Redebrandt H. (2022). Comparable Long-Term Tumor Control for Hypofractionated FLASH versus Conventional Radiation Therapy in an Immunocompetent Rat Glioma Model. Adv. Radiat. Oncol..

[4] Liljedahl E., Konradsson E., Linderfalk K., Gustafsson E., Petersson K., Ceberg C., Redebrandt H.N. (2023). Comparable Survival in Rats with Intracranial Glioblastoma Irradiated with Single-Fraction Conventional Radiotherapy or FLASH Radiotherapy. Front. Oncol..

[5] Iturri L., Bertho A., Lamirault C., Juchaux M., Gilbert C., Espenon J., Sebrie C., Jourdain L., Pouzoulet F., Verrelle P. (2023). Proton FLASH Radiation Therapy and Immune Infiltration: Evaluation in an Orthotopic Glioma Rat Model. Int. J. Radiat. Oncol. Biol. Phys..

[6] Iturri L., Bertho A., Lamirault C., Brisebard E., Juchaux M., Gilbert C., Espenon J., Sébrié C., Jourdain L., de Marzi L. (2023). Oxygen Supplementation in Anesthesia Can Block FLASH Effect and Anti-Tumor Immunity in Conventional Proton Therapy. Commun. Med. Lond..

[7] Dai Y., Liang R., Wang J., Zhang J., Wu D., Zhao R., Liu Z., Chen F. (2023). Fractionated FLASH Radiation in Xenografted Lung Tumors Induced FLASH Effect at a Split Dose of 2 Gy. Int. J. Radiat. Biol..

[8] Tinganelli W., Weber U., Puspitasari A., Simoniello P., Abdollahi A., Oppermann J., Schuy C., Horst F., Helm A., Fournier C. (2022). FLASH with Carbon Ions: Tumor Control, Normal Tissue Sparing, and Distal Metastasis in a Mouse Osteosarcoma Model. Radiother. Oncol..

[9] Tinganelli W., Puspitasari-Kokko A., Sokol O., Helm A., Simoniello P., Schuy C., Lerchl S., Eckert D., Oppermann J., Rehm A. (2025). FLASH Bragg-Peak Irradiation with a Therapeutic Carbon Ion Beam: First in Vivo Results. Int. J. Radiat. Oncol. Biol. Phys..

[10] Cao X., Zhang R., Esipova T.V., Allu S.R., Ashraf R., Rahman M., Gunn J.R., Bruza P., Gladstone D.J., Williams B.B. (2021). Quantification of Oxygen Depletion during FLASH Irradiation in Vitro and in Vivo. Int. J. Radiat. Oncol. Biol. Phys..

[11] Vilaplana-Lopera N., Kim J., Nam G., Tullis I.D.C., Paillas S., Ruan J.-L., Lee P.J., Jiang Y., Park S., Hou T. (2025). Tissue-Specific Iron Levels Modulate Lipid Peroxidation and the FLASH Radiotherapy Effect. Cell Death Dis..

[12] Almeida A., Togno M., Ballesteros-Zebadua P., Franco-Perez J., Geyer R., Schaefer R., Petit B., Grilj V., Meer D., Safai S. (2024). Dosimetric and Biologic Intercomparison between Electron and Proton FLASH Beams. Radiother. Oncol..

[13] Duval K.E.A., Aulwes E., Zhang R., Rahman M., Ashraf M.R., Sloop A., Sunnerberg J., Williams B.B., Cao X., Bruza P. (2023). Comparison of Tumor Control and Skin Damage in a Mouse Model after Ultra-High Dose Rate Irradiation and Conventional Irradiation. Radiat. Res..

[14] Padilla O., Minns H.E., Wei H.-J., Fan W., Webster-Carrion A., Tazhibi M., McQuillan N.M., Zhang X., Gallitto M., Yeh R. (2024). Immune Response Following FLASH and Conventional Radiation in Diffuse Midline Glioma. Int. J. Radiat. Oncol. Biol. Phys..

[15] Montay-Gruel P., Acharya M.M., Gonçalves Jorge P., Petit B., Petridis I.G., Fuchs P., Leavitt R., Petersson K., Gondré M., Ollivier J. (2021). Hypofractionated FLASH-RT as an Effective Treatment against Glioblastoma That Reduces Neurocognitive Side Effects in Mice. Clin. Cancer Res..

[16] Levy K., Natarajan S., Wang J., Chow S., Eggold J.T., Loo P.E., Manjappa R., Melemenidis S., Lartey F.M., Schüler E. (2020). Abdominal FLASH Irradiation Reduces Radiation-Induced Gastrointestinal Toxicity for the Treatment of Ovarian Cancer in Mice. Sci. Rep..

[17] Eggold J.T., Chow S., Melemenidis S., Wang J., Natarajan S., Loo P.E., Manjappa R., Viswanathan V., Kidd E.A., Engleman E. (2022). Abdominopelvic FLASH Irradiation Improves PD-1 Immune Checkpoint Inhibition in Preclinical Models of Ovarian Cancer. Mol. Cancer Ther..

[18] Liu K., Waldrop T., Aguilar E., Mims N., Neill D., Delahoussaye A., Li Z., Swanson D., Lin S.H., Koong A.C. (2025). Redefining FLASH Radiation Therapy: The Impact of Mean Dose Rate and Dose per Pulse in the Gastrointestinal Tract. Int. J. Radiat. Oncol. Biol. Phys..

[19] Zhu H., Xie D., Wang Y., Huang R., Chen X., Yang Y., Wang B., Peng Y., Wang J., Xiao D. (2023). Comparison of Intratumor and Local Immune Response between MV X-Ray FLASH and Conventional Radiotherapies. Clin. Transl. Radiat. Oncol..

[20] Almeida A., Godfroid C., Leavitt R.J., Montay-Gruel P., Petit B., Romero J., Ollivier J., Meziani L., Sprengers K., Paisley R. (2024). Antitumor Effect by Either FLASH or Conventional Dose Rate Irradiation Involves Equivalent Immune Responses. Int. J. Radiat. Oncol. Biol. Phys..

[21] Grilj V., Leavitt R.J., El Khatib M., Paisley R., Franco-Perez J., Petit B., Ballesteros-Zebadua P., Vozenin M.-C. (2024). In Vivo Measurements of Change in Tissue Oxygen Level during Irradiation Reveal Novel Dose Rate Dependence. Radiother. Oncol..

[22] Leavitt R.J., Almeida A., Grilj V., Montay-Gruel P., Godfroid C., Petit B., Bailat C., Limoli C.L., Vozenin M.-C. (2024). Acute Hypoxia Does Not Alter Tumor Sensitivity to FLASH Radiation Therapy. Int. J. Radiat. Oncol. Biol. Phys..

[23] Chabi S., Van To T.H., Leavitt R., Poglio S., Jorge P.G., Jaccard M., Petersson K., Petit B., Roméo P.-H., Pflumio F. (2021). Ultra-High-Dose-Rate FLASH and Conventional-Dose-Rate Irradiation Differentially Affect Human Acute Lymphoblastic Leukemia and Normal Hematopoiesis. Int. J. Radiat. Oncol. Biol. Phys..

[24] Dokic I., Moustafa M., Tessonnier T., Meister S., Ciamarone F., Akbarpour M., Krunic D., Haberer T., Debus J., Mairani A. (2025). Ultrahigh Dose Rate Helium Ion Beams: Minimizing Brain Tissue Damage While Preserving Tumor Control. Mol. Cancer Ther..

[25] Diffenderfer E.S., Verginadis I.I., Kim M.M., Shoniyozov K., Velalopoulou A., Goia D., Putt M., Hagan S., Avery S., Teo K. (2020). Design, Implementation, and in Vivo Validation of a Novel Proton FLASH Radiation Therapy System. Int. J. Radiat. Oncol. Biol. Phys..

[26] Sørensen B.S., Sitarz M.K., Ankjærgaard C., Johansen J.G., Andersen C.E., Kanouta E., Grau C., Poulsen P. (2022). Pencil Beam Scanning Proton FLASH Maintains Tumor Control While Normal Tissue Damage Is Reduced in a Mouse Model. Radiother. Oncol..

[27] Ni H., Reitman Z.J., Zou W., Akhtar M.N., Paul R., Huang M., Zhang D., Zheng H., Zhang R., Ma R. (2025). FLASH Radiation Reprograms Lipid Metabolism and Macrophage Immunity and Sensitizes Medulloblastoma to CAR-T Cell Therapy. Nat. Cancer.

[28] Velalopoulou A., Karagounis I.V., Cramer G.M., Kim M.M., Skoufos G., Goia D., Hagan S., Verginadis I.I., Shoniyozov K., Chiango J. (2021). FLASH Proton Radiotherapy Spares Normal Epithelial and Mesenchymal Tissues While Preserving Sarcoma Response. Cancer Res..

[29] Chowdhury P., Velalopoulou A., Verginadis I.I., Morcos G., Loo P.E., Kim M.M., Motlagh S.A.O., Shoniyozov K., Diffenderfer E.S., Ocampo E.A. (2024). Proton FLASH Radiotherapy Ameliorates Radiation-Induced Salivary Gland Dysfunction and Oral Mucositis and Increases Survival in a Mouse Model of Head and Neck Cancer. Mol. Cancer Ther..

[30] Gao F., Yang Y., Zhu H., Wang J., Xiao D., Zhou Z., Dai T., Zhang Y., Feng G., Li J. (2022). First Demonstration of the FLASH Effect with Ultrahigh Dose Rate High-Energy X-Rays. Radiother. Oncol..

[31] Bourhis J., Sozzi W.J., Jorge P.G., Gaide O., Bailat C., Duclos F., Patin D., Ozsahin M., Bochud F., Germond J.-F. (2019). Treatment of a First Patient with FLASH-Radiotherapy. Radiother. Oncol..

[32] Gjaldbæk B.W., Arendt M.L., Konradsson E., Bastholm Jensen K., Bäck S.Å.J., Munck Af Rosenschöld P., Ceberg C., Petersson K., Børresen B. (2024). Long-Term Toxicity and Efficacy of FLASH Radiotherapy in Dogs with Superficial Malignant Tumors. Front. Oncol..

[33] Børresen B., Arendt M.L., Konradsson E., Bastholm Jensen K., Bäck S.Å., Munck Af Rosenschöld P., Ceberg C., Petersson K. (2023). Evaluation of Single-Fraction High Dose FLASH Radiotherapy in a Cohort of Canine Oral Cancer Patients. Front. Oncol..

[34] Rohrer Bley C., Wolf F., Gonçalves Jorge P., Grilj V., Petridis I., Petit B., Böhlen T.T., Moeckli R., Limoli C., Bourhis J. (2022). Dose- and Volume-Limiting Late Toxicity of FLASH Radiotherapy in Cats with Squamous Cell Carcinoma of the Nasal Planum and in Mini Pigs. Clin. Cancer Res..

[35] Vozenin M.-C., De Fornel P., Petersson K., Favaudon V., Jaccard M., Germond J.-F., Petit B., Burki M., Ferrand G., Patin D. (2019). The Advantage of FLASH Radiotherapy Confirmed in Mini-Pig and Cat-Cancer Patients. Clin. Cancer Res..

[36] Ruan J.-L., Lee C., Wouters S., Tullis I.D.C., Verslegers M., Mysara M., Then C.K., Smart S.C., Hill M.A., Muschel R.J. (2021). Irradiation at Ultra-High (FLASH) Dose Rates Reduces Acute Normal Tissue Toxicity in the Mouse Gastrointestinal System. Int. J. Radiat. Oncol. Biol. Phys..

[37] Allen B.D., Alaghband Y., Kramár E.A., Ru N., Petit B., Grilj V., Petronek M.S., Pulliam C.F., Kim R.Y., Doan N.-L. (2023). Elucidating the Neurological Mechanism of the FLASH Effect in Juvenile Mice Exposed to Hypofractionated Radiotherapy. Neuro Oncol..

[38] Tavakkoli A.D., Clark M.A., Kheirollah A., Sloop A.M., Soderholm H.E., Daniel N.J., Petusseau A.F., Huang Y.H., Thomas C.R., Jarvis L.A. (2024). Anesthetic Oxygen Use and Sex Are Critical Factors in the FLASH Sparing Effect. Adv. Radiat. Oncol..

[39] Drayson O.G.G., Melemenidis S., Katila N., Viswanathan V., Kramár E.A., Zhang R., Kim R., Ru N., Petit B., Dutt S. (2024). A Multi-Institutional Study to Investigate the Sparing Effect after Whole Brain Electron FLASH in Mice: Reproducibility and Temporal Evolution of Functional, Electrophysiological, and Neurogenic Endpoints. Radiother. Oncol..

[40] Lu H., Li M., Quan C., Li C., Li D., Li Z., Xu J., Zhang L., Liu Q., Dong G. (2025). FLASH Irradiation Modulates Immune Responses and Accelerates Lung Recovery: A Single-Cell Perspective. Adv. Sci. Weinh..

[41] Zhu H., Liu S., Qiu J., Hu A., Zhou W., Wang J., Gu W., Zhu Y., Zha H., Xiang R. (2025). Instantaneous Dose Rate as a Crucial Factor in Reducing Mortality and Normal Tissue Toxicities in Murine Total-Body Irradiation: A Comparative Study of Dose Rate Combinations. Mol. Med..

[42] Cuitiño M.C., Fleming J.L., Jain S., Cetnar A., Ayan A.S., Woollard J., Manring H., Meng W., McElroy J.P., Blakaj D.M. (2023). Comparison of Gonadal Toxicity of Single-Fraction Ultra-High Dose Rate and Conventional Radiation in Mice. Adv. Radiat. Oncol..

[43] Dickstein D.L., Zhang R., Ru N., Vozenin M.-C., Perry B.C., Wang J., Baulch J.E., Acharya M.M., Limoli C.L. (2025). Structural Plasticity of Pyramidal Cell Neurons Measured after FLASH and Conventional Dose-Rate Irradiation. Brain Struct. Funct..

[44] Martínez-Rovira I., Montay-Gruel P., Petit B., Leavitt R.J., González-Vegas R., Froidevaux P., Juchaux M., Prezado Y., Yousef I., Vozenin M.-C. (2024). Infrared Microspectroscopy to Elucidate the Underlying Biomolecular Mechanisms of FLASH Radiotherapy. Radiother. Oncol..

[45] Petusseau A.F., Clark M., Bruza P., Gladstone D., Pogue B.W. (2024). Intracellular Oxygen Transient Quantification in Vivo during Ultra-High Dose Rate FLASH Radiation Therapy. Int. J. Radiat. Oncol. Biol. Phys..

[46] Smyth L.M.L., Donoghue J.F., Ventura J.A., Livingstone J., Bailey T., Day L.R.J., Crosbie J.C., Rogers P.A.W. (2018). Comparative Toxicity of Synchrotron and Conventional Radiation Therapy Based on Total and Partial Body Irradiation in a Murine Model. Sci. Rep..

[47] Zhang Q., Gerweck L.E., Cascio E., Yang Q., Huang P., Niemierko A., Bertolet A., Nesteruk K.P., McNamara A., Schuemann J. (2023). Proton FLASH Effects on Mouse Skin at Different Oxygen Tensions. Phys. Med. Biol..

[48] Evans T., Cooley J., Wagner M., Yu T., Zwart T. (2022). Demonstration of the FLASH Effect within the Spread-out Bragg Peak after Abdominal Irradiation of Mice. Int. J. Part. Ther..

[49] Mascia A., McCauley S., Speth J., Nunez S.A., Boivin G., Vilalta M., Sharma R.A., Perentesis J.P., Sertorio M. (2024). Impact of Multiple Beams on the FLASH Effect in Soft Tissue and Skin in Mice. Int. J. Radiat. Oncol. Biol. Phys..

[50] Lim T.L., Morral C., Verginadis I.I., Kim K., Luo L., Foley C.J., Kim M.M., Li N., Yoshor B., Njah K. (2024). Early Inflammation and Interferon Signaling Direct Enhanced Intestinal Crypt Regeneration after Proton FLASH Radiotherapy. bioRxiv.

[51] Kristensen L., Poulsen P.R., Kanouta E., Rohrer S., Ankjærgaard C., Andersen C.E., Johansen J.G., Simeonov Y., Weber U., Grau C. (2024). Spread-out Bragg Peak FLASH: Quantifying Normal Tissue Toxicity in a Murine Model. Front. Oncol..

[52] Verginadis I.I., Velalopoulou A., Kim M.M., Kim K., Paraskevaidis I., Bell B., Oliaei Motlagh S.A., Karaj A., Banerjee E., Finesso G. (2025). FLASH Proton Reirradiation, with or without Hypofractionation, Reduces Chronic Toxicity in the Normal Murine Intestine, Skin, and Bone. Radiother. Oncol..

[53] Bell B.I., Velten C., Pennock M., Kang M., Tanaka K.E., Selvaraj B., Bookbinder A., Koba W., Vercellino J., English J. (2025). Whole Abdominal Pencil Beam Scanned Proton FLASH Increases Acute Lethality. Int. J. Radiat. Oncol. Biol. Phys..

[54] Zhang Q., Gerweck L.E., Cascio E., Gu L., Yang Q., Dong X., Huang P., Bertolet A., Nesteruk K.P., Sung W. (2023). Absence of Tissue-Sparing Effects in Partial Proton FLASH Irradiation in Murine Intestine. Cancers.

[55] Zhang Q., Cascio E., Li C., Yang Q., Gerweck L.E., Huang P., Gottschalk B., Flanz J., Schuemann J. (2020). FLASH Investigations Using Protons: Design of Delivery System, Preclinical Setup and Confirmation of FLASH Effect with Protons in Animal Systems. Radiat. Res..

[56] Liu K., Titt U., Esplen N., Connell L., Konradsson E., Yang M., Wang X., Takaoka T., Li Z., Koong A.C. (2025). Discordance in Acute Gastrointestinal Toxicity between Synchrotron-Based Proton and Linac-Based Electron Ultra-High Dose Rate Irradiation. Int. J. Radiat. Oncol. Biol. Phys..

[57] Shi X., Yang Y., Zhang W., Wang J., Xiao D., Ren H., Wang T., Gao F., Liu Z., Zhou K. (2022). FLASH X-Ray Spares Intestinal Crypts from Pyroptosis Initiated by cGAS-STING Activation upon Radioimmunotherapy. Proc. Natl. Acad. Sci. USA.

[58] Akulinichev S.V., Glukhov S.I., Kuznetsova E.A., Gavrilov Y.K., Kokontsev D.A., Martynova V.V., Merzlikin G.V., Yakovlev I.A. (2025). Manifestation of the FLASH Effect in Proton Irradiation of Embryos. Int. J. Radiat. Biol..

[59] Iwata H., Toshito T., Omachi C., Umezawa M., Yamada M., Tanaka K., Nakajima K., Tsuzuki Y., Matsumoto K., Kawai T. (2025). Proton FLASH Irradiation Using a Synchrotron Accelerator: Differences by Irradiation Positions. Int. J. Radiat. Oncol. Biol. Phys..

[60] Karsch L., Pawelke J., Brand M., Hans S., Hideghéty K., Jansen J., Lessmann E., Löck S., Schürer M., Schurig R. (2022). Beam Pulse Structure and Dose Rate as Determinants for the Flash Effect Observed in Zebrafish Embryo. Radiother. Oncol..

[61] Pawelke J., Brand M., Hans S., Hideghéty K., Karsch L., Lessmann E., Löck S., Schürer M., Szabó E.R., Beyreuther E. (2021). Electron Dose Rate and Oxygen Depletion Protect Zebrafish Embryos from Radiation Damage. Radiother. Oncol..

[62] Ghannam Y., Chiavassa S., Saade G., Koumeir C., Blain G., Delpon G., Evin M., Haddad F., Maigne L., Mouchard Q. (2023). First Evidence of in Vivo Effect of FLASH Radiotherapy with Helium Ions in Zebrafish Embryos. Radiother. Oncol..

[63] Beyreuther E., Brand M., Hans S., Hideghéty K., Karsch L., Leßmann E., Schürer M., Szabó E.R., Pawelke J. (2019). Feasibility of Proton FLASH Effect Tested by Zebrafish Embryo Irradiation. Radiother. Oncol..

[64] Saade G., Bogaerts E., Chiavassa S., Blain G., Delpon G., Evin M., Ghannam Y., Haddad F., Haustermans K., Koumeir C. (2023). Ultrahigh-Dose-Rate Proton Irradiation Elicits Reduced Toxicity in Zebrafish Embryos. Adv. Radiat. Oncol..

[65] Horst F., Bodenstein E., Brand M., Hans S., Karsch L., Lessmann E., Löck S., Schürer M., Pawelke J., Beyreuther E. (2024). Dose and Dose Rate Dependence of the Tissue Sparing Effect at Ultra-High Dose Rate Studied for Proton and Electron Beams Using the Zebrafish Embryo Model. Radiother. Oncol..

[66] Hart A., Dudzic J.P., Clarke J.W., Eby J., Perlman S.J., Bazalova-Carter M. (2024). High-Throughput, Low-Cost FLASH: Irradiation of Drosophila Melanogaster with Low-Energy X-Rays Using Time Structures Spanning Conventional and Ultrahigh Dose Rates. J. Radiat. Res..

[67] Schoenauen L., Stubbe F.-X., Van Gestel D., Penninckx S., Heuskin A.-C.C. (2024). Elegans: A Potent Model for High-Throughput Screening Experiments Investigating the FLASH Effect. Clin. Transl. Radiat. Oncol..

[68] Fouillade C., Curras-Alonso S., Giuranno L., Quelennec E., Heinrich S., Bonnet-Boissinot S., Beddok A., Leboucher S., Karakurt H.U., Bohec M. (2020). FLASH Irradiation Spares Lung Progenitor Cells and Limits the Incidence of Radio-Induced Senescence. Clin. Cancer Res..

[69] Dokic I., Meister S., Bojcevski J., Tessonnier T., Walsh D., Knoll M., Mein S., Tang Z., Vogelbacher L., Rittmueller C. (2022). Neuroprotective Effects of Ultra-High Dose Rate FLASH Bragg Peak Proton Irradiation. Int. J. Radiat. Oncol. Biol. Phys..

[70] Montay-Gruel P., Acharya M.M., Petersson K., Alikhani L., Yakkala C., Allen B.D., Ollivier J., Petit B., Jorge P.G., Syage A.R. (2019). Long-Term Neurocognitive Benefits of FLASH Radiotherapy Driven by Reduced Reactive Oxygen Species. Proc. Natl. Acad. Sci. USA.

[71] Barghouth P.G., Melemenidis S., Montay-Gruel P., Ollivier J., Viswanathan V., Jorge P.G., Soto L.A., Lau B.C., Sadeghi C., Edlabadkar A. (2023). FLASH-RT Does Not Affect Chromosome Translocations and Junction Structures beyond That of CONV-RT Dose-Rates. Radiother. Oncol..

[72] Buonanno M., Grilj V., Brenner D.J. (2019). Biological Effects in Normal Cells Exposed to FLASH Dose Rate Protons. Radiother. Oncol..

[73] Matsuura T., Egashira Y., Nishio T., Matsumoto Y., Wada M., Koike S., Furusawa Y., Kohno R., Nishioka S., Kameoka S. (2010). Apparent Absence of a Proton Beam Dose Rate Effect and Possible Differences in RBE between Bragg Peak and Plateau. Med. Phys..

[74] Tashiro M., Yoshida Y., Oike T., Nakao M., Yusa K., Hirota Y., Ohno T. (2022). First Human Cell Experiments with FLASH Carbon Ions. Anticancer. Res..

[75] Akulinichev S.V., Gavrilov Y.K., Glukhov S.I., Ivanov A.V., Kokontsev D.A., Kulinich T.M., Kuznetsova E.A., Martynova V.V., Yakovlev I.A. (2023). Analysis of Cell Response to Ultrahigh Dose-Rate Proton Irradiation. Bull. Russ. Acad. Sci. Phys..

[76] Guo Z., Buonanno M., Harken A., Zhou G., Hei T.K. (2022). Mitochondrial Damage Response and Fate of Normal Cells Exposed to FLASH Irradiation with Protons. Radiat. Res..

[77] Liljedahl E., Konradsson E., Gustafsson E., Jonsson K.F., Olofsson J.K., Ceberg C., Redebrandt H.N. (2022). Long-Term Anti-Tumor Effects Following Both Conventional Radiotherapy and FLASH in Fully Immunocompetent Animals with Glioblastoma. Sci. Rep..

[78] Shukla S., Saha T., Rama N., Acharya A., Le T., Bian F., Donovan J., Tan L.A., Vatner R., Kalinichenko V. (2023). Ultra-High Dose-Rate Proton FLASH Improves Tumor Control. Radiother. Oncol..

[79] Tobias Böhlen T., Psoroulas S., Aylward J.D., Beddar S., Douralis A., Delpon G., Garibaldi C., Gasparini A., Schüler E., Stephan F. (2024). Recording and Reporting of Ultra-High Dose Rate “FLASH” Delivery for Preclinical and Clinical Settings. Radiother. Oncol..

[80] Chaikh A., Édouard M., Huet C., Milliat F., Villagrasa C., Isambert A. (2024). Towards Clinical Application of Ultra-High Dose Rate Radiotherapy and the FLASH Effect: Challenges and Current Status. Cancer Radiother..

[81] DeFrancisco J., Kim S. (2025). A Systematic Review of Electron FLASH Dosimetry and Beam Control Mechanisms Utilized with Modified Non-Clinical LINACs. J. Appl. Clin. Med. Phys..

[82] Subiel A., Bourgouin A., Kranzer R., Peier P., Frei F., Gomez F., Knyziak A., Fleta C., Bailat C., Schüller A. (2024). Metrology for Advanced Radiotherapy Using Particle Beams with Ultra-High Dose Rates. Phys. Med. Biol..

[83] Atkinson J., Bezak E., Le H., Kempson I. (2023). The Current Status of FLASH Particle Therapy: A Systematic Review. Phys. Eng. Sci. Med..

[84] Diffenderfer E.S., Sørensen B.S., Mazal A., Carlson D.J. (2022). The Current Status of Preclinical Proton FLASH Radiation and Future Directions. Med. Phys..

[85] Dosanjh M., Degiovanni A., Necchi M.M., Benedetto E. (2025). Multidisciplinary Collaboration and Novel Technological Advances in Hadron Therapy. Technol. Cancer Res. Treat..

[86] Laurent P.-A., André F., Bobard A., Deandreis D., Demaria S., Depil S., Eichmüller S.B., Fernandez-Palomo C., Foijer F., Galluzzi L. (2025). Pushing the Boundaries of Radiotherapy-Immunotherapy Combinations: Highlights from the 7th Immunorad Conference. Oncoimmunology.

[87] Metzkes-Ng J., Brack F.-E., Kroll F., Bernert C., Bock S., Bodenstein E., Brand M., Cowan T.E., Gebhardt R., Hans S. (2023). The DRESDEN PLATFORM Is a Research Hub for Ultra-High Dose Rate Radiobiology. Sci. Rep..

[88] Snider J.W., Mayr N.A., Molitoris J., Chhabra A.M., Mossahebi S., Griffin R., Mohiuddin M., Zhang H., Amendola B., Tubin S. (2025). The Radiosurgery Society Working Groups on GRID, LATTICE, Microbeam, and FLASH Radiotherapies: Advancements Symposium and Subsequent Progress Made. Prac. Radiat. Oncol..

[89] Plante I., Cucinotta F.A. (2015). Simulation of the Radiolysis of Water Using Green’s Functions of the Diffusion Equation. Radiat. Prot. Dosim..

[90] Elkins M.H., Williams H.L., Shreve A.T., Neumark D.M. (2013). Relaxation Mechanism of the Hydrated Electron. Science.

[91] Loh Z.-H., Doumy G., Arnold C., Kjellsson L., Southworth S.H., Al Haddad A., Kumagai Y., Tu M.-F., Ho P.J., March A.M. (2020). Observation of the Fastest Chemical Processes in the Radiolysis of Water. Science.

[92] Sies H., Berndt C., Jones D.P. (2017). Oxidative Stress. Annu. Rev. Biochem..

[93] Jay-Gerin J.-P. (2020). Ultra-High Dose-Rate (FLASH) Radiotherapy: Generation of Early, Transient, Strongly Acidic Spikes in the Irradiated Tumor Environment. Cancer Radiother..

[94] Grimes D.R., Warren D.R., Warren S. (2017). Hypoxia Imaging and Radiotherapy: Bridging the Resolution Gap. Br. J. Radiol..

[95] Jansen J., Knoll J., Beyreuther E., Pawelke J., Skuza R., Hanley R., Brons S., Pagliari F., Seco J. (2021). Does FLASH Deplete Oxygen? Experimental Evaluation for Photons, Protons, and Carbon Ions. Med. Phys..

[96] Karle C., Liew H., Tessonnier T., Mein S., Petersson K., Schömers C., Scheloske S., Brons S., Cee R., Major G. (2025). Oxygen Consumption Measurements at Ultra-High Dose Rate over a Wide LET Range. Med. Phys..

[97] Van Slyke A.L., El Khatib M., Velalopoulou A., Diffenderfer E., Shoniyozov K., Kim M.M., Karagounis I.V., Busch T.M., Vinogradov S.A., Koch C.J. (2022). Oxygen Monitoring in Model Solutions and in Vivo in Mice during Proton Irradiation at Conventional and FLASH Dose Rates. Radiat. Res..

[98] Kanouta E., Johansen J.G., Poulsen S., Kristensen L., Sørensen B.S., Grau C., Busk M., Poulsen P.R. (2024). Correlation between Local Instantaneous Dose Rate and Oxygen Pressure Reduction during Proton Pencil Beam Scanning Irradiation. Phys. Imaging Radiat. Oncol..

[99] El Khatib M., Motlagh A.O., Beyer J.N., Troxler T., Allu S.R., Sun Q., Burslem G.M., Vinogradov S.A. (2024). Direct Measurements of FLASH-Induced Changes in Intracellular Oxygenation. Int. J. Radiat. Oncol. Biol. Phys..

[100] Clark M.A., Tavakkoli A.D., Petusseau A.F., Scorzo A.V., Kheirollah A., Strawbridge R.R., Davis S.C., Bruza P., Pogue B.W., Gladstone D.J. (2025). Dynamic Oxygen Assessment Techniques Enable Determination of Anesthesia’s Impact on Tissue. Sci. Rep..

[101] Sunnerberg J.P., Hunter D.I., Sloop A.M., Tavakkoli A.D., Bruza P., Zhang R., Gui J., Jarvis L.A., Swartz H.M., Gladstone D.J. (2025). Timescale of FLASH Sparing Effect Determined by Varying Temporal Split of Dose Delivery in Mice. Int. J. Radiat. Oncol..

[102] Sunnerberg J.P., Zhang R., Gladstone D.J., Swartz H.M., Gui J., Pogue B.W. (2023). Mean Dose Rate in Ultra-High Dose Rate Electron Irradiation Is a Significant Predictor for O_2_ consumption and H_2_O_2_ yield. Phys. Med. Biol..

[103] Pétusseau A., Bruza P., Pogue B. (2022). Protoporphyrin IX Delayed Fluorescence Imaging: A Modality for Hypoxia-Based Surgical Guidance. J. Biomed. Opt..

[104] Kyle A.H., Karan T., Baker J.H.E., Püspöky Banáth J., Wang T., Liu A., Mendez C., Peter Petric M., Duzenli C., Minchinton A.I. (2024). Detection of FLASH-Radiotherapy Tissue Sparing in a 3D-Spheroid Model Using DNA Damage Response Markers. Radiother. Oncol..

[105] Minami K., Yagi M., Fujita K., Nagata K., Hidani R., Hamatani N., Tsubouchi T., Takashina M., Umezawa M., Nomura T. (2025). The Appropriate Conditions for the Cell Sparing (FLASH) Effect Exist in Ultra-High Dose Rate Carbon Ion Irradiation. Anticancer Res..

[106] Chaoui M., Tayalati Y., Bouhali O., Ramos-Méndez J. (2025). Monte Carlo Track-Structure Simulation of the Impact of Ultra-Hight Dose Rate and Oxygen Concentration on the Fenton Reaction. bioRxiv.

[107] Tessonnier T., Mein S., Walsh D.W.M., Schuhmacher N., Liew H., Cee R., Galonska M., Scheloske S., Schömers C., Weber U. (2021). FLASH Dose Rate Helium Ion Beams: First in Vitro Investigations. Int. J. Radiat. Oncol. Biol. Phys..

[108] Tinganelli W., Sokol O., Quartieri M., Puspitasari A., Dokic I., Abdollahi A., Durante M., Haberer T., Debus J., Boscolo D. (2022). Ultra-High Dose Rate (FLASH) Carbon Ion Irradiation: Dosimetry and First Cell Experiments. Int. J. Radiat. Oncol. Biol. Phys..

[109] D-Kondo J.N., Borys D., Ruciński A., Brzozowska B., Masilela T.A.M., Grochowska-Tatarczak M., Węgrzyn M., Ramos-Mendez J. (2025). Effect of FLASH Dose-Rate and Oxygen Concentration in the Production of H_2_O_2_ in Cellular-like Media versus Water: A Monte Carlo Track-Structure Study. Phys. Med. Biol..

[110] Guo L., Medin P.M., Wang K.K.-H. (2024). A Microscopic Oxygen Transport Model for Ultra-High Dose Rate Radiotherapy in Vivo: The Impact of Physiological Conditions on FLASH Effect. Med. Phys..

[111] Rabeya I., Meesungnoen J., Jay-Gerin J.-P. (2025). Oxygen Depletion and the Role of Cellular Antioxidants in FLASH Radiotherapy: Mechanistic Insights from Monte Carlo Radiation-Chemical Modeling. Antioxidants.

[112] Taylor E., Hill R.P., Létourneau D. (2022). Modeling the Impact of Spatial Oxygen Heterogeneity on Radiolytic Oxygen Depletion during FLASH Radiotherapy. Phys. Med. Biol..

[113] Taylor E., Létourneau D. (2024). How Quickly Does FLASH Need to Be Delivered? A Theoretical Study of Radiolytic Oxygen Depletion Kinetics in Tissues. Phys. Med. Biol..

[114] Daugherty E.C., Zhang Y., Xiao Z., Mascia A.E., Sertorio M., Woo J., McCann C., Russell K.J., Sharma R.A., Khuntia D. (2024). FLASH Radiotherapy for the Treatment of Symptomatic Bone Metastases in the Thorax (FAST-02): Protocol for a Prospective Study of a Novel Radiotherapy Approach. Radiat. Oncol..

[115] Daugherty E.C., Mascia A., Zhang Y., Lee E., Xiao Z., Sertorio M., Woo J., McCann C., Russell K., Levine L. (2023). FLASH Radiotherapy for the Treatment of Symptomatic Bone Metastases (FAST-01): Protocol for the First Prospective Feasibility Study. JMIR Res. Protoc..

[116] Perstin A., Poirier Y., Sawant A., Tambasco M. (2022). Quantifying the DNA-Damaging Effects of FLASH Irradiation with Plasmid DNA. Int. J. Radiat. Oncol. Biol. Phys..

[117] Gupta S., Inman J.L., Chant J.D., Obst-Huebl L., Nakamura K., Costello S.M., Marqusee S., Mao J.-H., Kunz L., Paisley R. (2023). A Novel Platform for Evaluating Dose Rate Effects on Oxidative Damage to Peptides: Toward a High-Throughput Method to Characterize the Mechanisms Underlying the FLASH Effect. Radiat. Res..

[118] Kusumoto T., Inaniwa T., Mizushima K., Sato S., Hojo S., Kitamura H., Konishi T., Kodaira S. (2022). Radiation Chemical Yields of 7-Hydroxy-Coumarin-3-Carboxylic Acid for Proton- and Carbon-Ion Beams at Ultra-High Dose Rates: Potential Roles in FLASH Effects. Radiat. Res..

[119] Bedford J.L. (2025). Pulse-by-Pulse Treatment Planning and Its Application to Generic Observations of Ultra-High Dose Rate (FLASH) Radiotherapy with Photons and Protons. Phys. Med. Biol..

[120] Castelli L., Camazzola G., Fuss M.C., Boscolo D., Krämer M., Tozzini V., Durante M., Scifoni E. (2025). Probing Spatiotemporal Effects of Intertrack Recombination with a New Implementation of Simultaneous Multiple Tracks in TRAX-CHEM. Int. J. Mol. Sci..

[121] Chaoui M., Bouhali O., Tayalati Y. (2025). FLASH Radiotherapy: Technical Advances, Evidence of the FLASH Effect and Mechanistic Insights. Biomed. Phys. Eng. Express.

[122] Cucinotta F.A., Smirnova O.A. (2024). Effects of Partial-Body, Continuous/Pulse Irradiation at Dose Rates from FLASH to Conventional Rates on the Level of Surviving Blood Lymphocytes: Modeling Approach II. Two- and Multiple-Pulse Irradiation. Radiat. Res..

[123] González-Crespo I., Gómez F., López Pouso Ó., Pardo-Montero J. (2024). An In-Silico Study of Conventional and FLASH Radiotherapy Iso-Effectiveness: Potential Impact of Radiolytic Oxygen Depletion on Tumor Growth Curves and Tumor Control Probability. Phys. Med. Biol..

[124] Ma J., Lin Y., Tang M., Zhu Y.-N., Gan G.N., Rotondo R.L., Chen R.C., Gao H. (2024). Simultaneous Dose and Dose Rate Optimization via Dose Modifying Factor Modeling for FLASH Effective Dose. Med. Phys..

[125] Penabeï S., Meesungnoen J., Jay-Gerin J.-P. (2024). Comparative Analysis of Cystamine and Cysteamine as Radioprotectors and Antioxidants: Insights from Monte Carlo Chemical Modeling under High Linear Energy Transfer Radiation and High Dose Rates. Int. J. Mol. Sci..

[126] Pikes G., Dass J., Gill S., Ebert M., Reynolds M., Rowshanfarzad P. (2025). Monte Carlo in the Mechanistic Modelling of the FLASH Effect: A Review. Phys. Med. Biol..

[127] Shiraishi Y., Matsuya Y., Fukunaga H. (2024). Possible Mechanisms and Simulation Modeling of FLASH Radiotherapy. Radiol. Phys. Technol..

[128] Lai Y., Jia X., Chi Y. (2021). Modeling the Effect of Oxygen on the Chemical Stage of Water Radiolysis Using GPU-Based Microscopic Monte Carlo Simulations, with an Application in FLASH Radiotherapy. Phys. Med. Biol..

[129] Peng Y., Lai Y., Yin L., Chi Y., Li H., Jia X. (2025). Investigating Radical Yield Variations in FLASH and Conventional Proton Irradiation via Microscopic Monte Carlo Simulations. Phys. Med. Biol..

[130] Shin W.-G., Ramos-Mendez J., Tran N.H., Okada S., Perrot Y., Villagrasa C., Incerti S. (2021). Geant4-DNA Simulation of the Pre-Chemical Stage of Water Radiolysis and Its Impact on Initial Radiochemical Yields. Phys. Med..

[131] Blain G., Vandenborre J., Villoing D., Fiegel V., Fois G.R., Haddad F., Koumeir C., Maigne L., Métivier V., Poirier F. (2022). Proton Irradiations at Ultra-High Dose Rate vs. Conventional Dose Rate: Strong Impact on Hydrogen Peroxide Yield. Radiat. Res..

[132] Matsumoto K.-I., Ueno M., Nyui M., Shoji Y., Nakanishi I. (2021). Effects of LET on Oxygen-Dependent and-Independent Generation of Hydrogen Peroxide in Water Irradiated by Carbon-Ion Beams. Free Radic. Res..

[133] Zhang T., Stengl C., Derksen L., Palskis K., Koritsidis K., Zink K., Adeberg S., Major G., Weishaar D., Theiß U. (2024). Analysis of Hydrogen Peroxide Production in Pure Water: Ultrahigh versus Conventional Dose-Rate Irradiation and Mechanistic Insights. Med. Phys..

[134] Domnanich K.A., Severin G.W. (2022). A Model for Radiolysis in a Flowing-Water Target during High-Intensity Proton Irradiation. ACS Omega.

[135] Kusumoto T., Danvin A., Mamiya T., Arnone A., Chefson S., Galindo C., Peaupardin P., Raffy Q., Kamiguchi N., Amano D. (2024). Dose Rate Effects on Hydrated Electrons, Hydrogen Peroxide, and a OH Radical Molecular Probe under Clinical Energy Protons. Radiat. Res..

[136] Kusumoto T., Kitamura H., Hojo S., Konishi T., Kodaira S. (2020). Significant Changes in Yields of 7-Hydroxy-Coumarin-3-Carboxylic Acid Produced under FLASH Radiotherapy Conditions. RSC Adv..

[137] Pastina B., LaVerne J.A. (2001). Effect of Molecular Hydrogen on Hydrogen Peroxide in Water Radiolysis. J. Phys. Chem. A.

[138] Cao X., Parks A., Thomas W., Reed M.S., Culberson W.S., Pogue B.W. (2025). Hydrated Electron Yield Dependence on Instantaneous Dose Rates with Electron Ultra-High Dose Rate (UHDR) Irradiation. Phys. Med. Biol..

[139] Adrian G., Konradsson E., Lempart M., Bäck S., Ceberg C., Petersson K. (2020). The FLASH Effect Depends on Oxygen Concentration. Br. J. Radiol..

[140] Cooper C.R., Jones D., Jones G.D., Petersson K. (2022). FLASH Irradiation Induces Lower Levels of DNA Damage Ex Vivo, an Effect Modulated by Oxygen Tension, Dose, and Dose Rate. Br. J. Radiol..

[141] Alanazi A., Meesungnoen J., Jay-Gerin J.-P. (2020). A Computer Modeling Study of Water Radiolysis at High Dose Rates. Relevance to FLASH Radiotherapy. Radiat. Res..

[142] Nicholls P. (2007). The Oxygenase-Peroxidase Theory of Bach and Chodat and Its Modern Equivalents: Change and Permanence in Scientific Thinking as Shown by Our Understanding of the Roles of Water, Peroxide, and Oxygen in the Functioning of Redox Enzymes. Biochem. Mosc..

[143] Azzam E.I., Jay-Gerin J.-P., Pain D. (2012). Ionizing Radiation-Induced Metabolic Oxidative Stress and Prolonged Cell Injury. Cancer Lett..

[144] Pak V.V., Ezeriņa D., Lyublinskaya O.G., Pedre B., Tyurin-Kuzmin P.A., Mishina N.M., Thauvin M., Young D., Wahni K., Martínez Gache S.A. (2020). Ultrasensitive Genetically Encoded Indicator for Hydrogen Peroxide Identifies Roles for the Oxidant in Cell Migration and Mitochondrial Function. Cell Metab..

[145] Roscoe J.M., Sevier C.S. (2020). Pathways for Sensing and Responding to Hydrogen Peroxide at the Endoplasmic Reticulum. Cells.

[146] Kunz L.V., Schaefer R., Kacem H., Ollivier J., Togno M., Chappuis F., Weber D., Lomax A., Limoli C.L., Psoroulas S. (2025). Plasmid DNA Strand Breaks Are Dose Rate Independent at Clinically Relevant Proton Doses and under Biological Conditions. Radiat. Res..

[147] Kuipers M.E., van Liefferinge F., van der Wal E., Rovituso M., Slats A.M., Hiemstra P.S., Van Doorn-Wink K.C.J. (2025). Effect of FLASH Proton Therapy on Primary Bronchial Epithelial Cell Organoids. Clin. Transl. Radiat. Oncol..

[148] Sunnerberg J.P., Tavakkoli A.D., Petusseau A.F., Daniel N.J., Sloop A.M., Schreiber W.A., Gui J., Zhang R., Swartz H.M., Hoopes P.J. (2025). Oxygen Consumption in Vivo by Ultra-High Dose Rate Electron Irradiation Depends upon Baseline Tissue Oxygenation. Int. J. Radiat. Oncol. Biol. Phys..

[149] Rudigkeit S., Schmid T.E., Dombrowsky A.C., Stolz J., Bartzsch S., Chen C.-B., Matejka N., Sammer M., Bergmaier A., Dollinger G. (2024). Proton-FLASH: Effects of Ultra-High Dose Rate Irradiation on an in-Vivo Mouse Ear Model. Sci. Rep..

[150] Roy T.K., Secomb T.W. (2021). Effects of Impaired Microvascular Flow Regulation on Metabolism-perfusion Matching and Organ Function. Microcirculation.

[151] El Khatib M., Van Slyke A.L., Velalopoulou A., Kim M.M., Shoniyozov K., Allu S.R., Diffenderfer E.E., Busch T.M., Wiersma R.D., Koch C.J. (2022). Ultrafast Tracking of Oxygen Dynamics during Proton FLASH. Int. J. Radiat. Oncol. Biol. Phys..

[152] Spitz D.R., Buettner G.R., Petronek M.S., St-Aubin J.J., Flynn R.T., Waldron T.J., Limoli C.L. (2019). An Integrated Physico-Chemical Approach for Explaining the Differential Impact of FLASH versus Conventional Dose Rate Irradiation on Cancer and Normal Tissue Responses. Radiother. Oncol..

[153] Secomb T.W., Hsu R., Dewhirst M.W., Klitzman B., Gross J.F. (1993). Analysis of Oxygen Transport to Tumor Tissue by Microvascular Networks. Int. J. Radiat. Oncol..

[154] Tannock I.F. (1972). Oxygen Diffusion and the Distribution of Cellular Radiosensitivity in Tumours. Br. J. Radiol..

[155] Groebe K., Vaupel P. (1988). Evaluation of Oxygen Diffusion Distances in Human Breast Cancer Xenografts Using Tumor-Specific in Vivo Data: Role of Various Mechanisms in the Development of Tumor Hypoxia. Int. J. Radiat. Oncol..

[156] Dewhirst M.W. (2009). Relationships between Cycling Hypoxia, HIF-1, Angiogenesis and Oxidative Stress. Radiat. Res..

[157] Hong B.-J., Kim J., Jeong H., Bok S., Kim Y.-E., Ahn G.-O. (2016). Tumor Hypoxia and Reoxygenation: The Yin and Yang for Radiotherapy. Radiat. Oncol. J..

[158] Geirnaert F., Kerkhove L., Montay-Gruel P., Gevaert T., Dufait I., De Ridder M. (2025). Exploring the Metabolic Impact of FLASH Radiotherapy. Cancers.

[159] Nakamura H., Takada K. (2021). Reactive Oxygen Species in Cancer: Current Findings and Future Directions. Cancer Sci..

[160] Han J., Mei Z., Lu C., Qian J., Liang Y., Sun X., Pan Z., Kong D., Xu S., Liu Z. (2021). Ultra-High Dose Rate FLASH Irradiation Induced Radio-Resistance of Normal Fibroblast Cells Can Be Enhanced by Hypoxia and Mitochondrial Dysfunction Resulting from Loss of Cytochrome C. Front. Cell Dev. Biol..

[161] Parajuli R.K., Sano H., Ueno M., Gao S., Suzuki S., Sumiyoshi A., Osada K., Obata T., Aoki I., Matsumoto K. (2025). Quantification of Oxygenation and Oxygen Consumption Rates in the Mouse Brain Based on Tissue Oxygen Level-Dependent (TOLD) MRI. NMR Biomed..

[162] Brizel D., Lin S., Johnson J., Brooks J., Dewhirst M., Piantadosi C. (1995). The Mechanisms by Which Hyperbaric Oxygen and Carbogen Improve Tumour Oxygenation. Br. J. Cancer.

[163] Sørensen B.S., Kanouta E., Ankjærgaard C., Kristensen L., Johansen J.G., Sitarz M.K., Andersen C.E., Grau C., Poulsen P. (2024). Proton FLASH: Impact of Dose Rate and Split Dose on Acute Skin Toxicity in a Murine Model. Int. J. Radiat. Oncol. Biol. Phys..

[164] Town C.D. (1967). Effect of High Dose Rates on Survival of Mammalian Cells. Nature.

[165] Hassannia B., Vandenabeele P., Vanden Berghe T. (2019). Targeting Ferroptosis to Iron out Cancer. Cancer Cell.

[166] Cooper C.R., Jones D.J.L., Jones G.D.D., Petersson K. (2023). Comet Assay Profiling of FLASH-Induced Damage: Mechanistic Insights into the Effects of FLASH Irradiation. Int. J. Mol. Sci..

[167] Soto L.A., Casey K.M., Wang J., Blaney A., Manjappa R., Breitkreutz D., Skinner L., Dutt S., Ko R.B., Bush K. (2020). FLASH Irradiation Results in Reduced Severe Skin Toxicity Compared to Conventional-Dose-Rate Irradiation. Radiat. Res..

[168] Portier L., Daira P., Fourmaux B., Heinrich S., Becerra M., Fouillade C., Berthault N., Dutreix M., Londoño-Vallejo A., Verrelle P. (2024). Differential Remodeling of the Oxylipin Pool after FLASH versus Conventional Dose-Rate Irradiation in Vitro and in Vivo. Int. J. Radiat. Oncol. Biol. Phys..

[169] Vaupel P., Multhoff G. (2021). Revisiting the Warburg Effect: Historical Dogma *versus* Current Understanding. J. Physiol..

[170] Lu J., Tan M., Cai Q. (2015). The Warburg Effect in Tumor Progression: Mitochondrial Oxidative Metabolism as an Anti-Metastasis Mechanism. Cancer Lett..

[171] Xu M., Peng Q., Zhang J., Xu Z., Cheng X., Cao Z., Zhang Y. (2025). Comparative Transcriptomic Analysis Unveils Divergent Effects of FLASH versus Conventional Irradiation on Skin Cells. Dose Response.

[172] Li M., Zhang L., Gao A., Xu J., Wang X., Liu X., Yan D., Zou D., Wu S., Sun B. (2025). Molecular Mechanisms of Lung Injury from Ultra High and Conventional Dose Rate Pulsed Radiation Based on 4D DIA Proteomics Study. Sci. Rep..

[173] Ford N.L., Ren X., Egoriti L., Esplen N., Radel S., Humphries B., Koay H.-W., Planche T., Hoehr C., Gottberg A. (2025). Respiratory-Gated Micro-Computed Tomography Imaging to Measure Radiation-Induced Lung Injuries in Mice Following Ultra-High Dose-Rate and Conventional Dose-Rate Radiation Therapy. J. Med. Imaging.

[174] Mohamad O., Sishc B., Saha J., Pompos A., Rahimi A., Story M., Davis A., Kim D.W. (2017). Carbon Ion Radiotherapy: A Review of Clinical Experiences and Preclinical Research, with an Emphasis on DNA Damage/Repair. Cancers.

[175] Kim K., Kim M.M., Skoufos G., Diffenderfer E.S., Motlagh S.A.O., Kokkorakis M., Koliaki I., Morcos G., Shoniyozov K., Griffin J. (2024). FLASH Proton Radiation Therapy Mitigates Inflammatory and Fibrotic Pathways and Preserves Cardiac Function in a Preclinical Mouse Model of Radiation-Induced Heart Disease. Int. J. Radiat. Oncol. Biol. Phys..

[176] Kim M.M., Verginadis I.I., Goia D., Haertter A., Shoniyozov K., Zou W., Maity A., Busch T.M., Metz J.M., Cengel K.A. (2021). Comparison of FLASH Proton Entrance and the Spread-out Bragg Peak Dose Regions in the Sparing of Mouse Intestinal Crypts and in a Pancreatic Tumor Model. Cancers.

[177] Katsuki S., Minami K., Oniwa K., Yagi M., Shimizu S., Hamatani N., Takashina M., Kanai T., Ogawa K. (2025). Ultra-High Dose Rate (FLASH) Carbon Ion Irradiation Inhibited Immune Suppressive Protein Expression on Pan02 Cell Line. J. Radiat. Res..

[178] Kristensen L., Rohrer S., Hoffmann L., Præstegaard L.H., Ankjærgaard C., Andersen C.E., Kanouta E., Johansen J.G., Sahlertz M., Nijkamp J. (2025). Electron vs Proton FLASH Radiation on Murine Skin Toxicity. Radiother. Oncol..

[179] Cunningham S., McCauley S., Vairamani K., Speth J., Girdhani S., Abel E., Sharma R.A., Perentesis J.P., Wells S.I., Mascia A. (2021). FLASH Proton Pencil Beam Scanning Irradiation Minimizes Radiation-Induced Leg Contracture and Skin Toxicity in Mice. Cancers.

[180] Böhlen T.T., Zeverino M., Germond J.-F., Kinj R., Schiappacasse L., Bochud F., Herrera F., Bourhis J., Moeckli R. (2024). Hybrid Ultra-High and Conventional Dose Rate Treatments with Electrons and Photons for the Clinical Transfer of FLASH-RT to Deep-Seated Targets: A Treatment Planning Study. Radiother. Oncol..

[181] Wang Y., Qi S.-N., Bi N., Li Y.-X. (2025). FLASH Radiotherapy Combined with Immunotherapy: From Biological Mechanisms to Blockbuster Therapeutics. Transl. Oncol..

[182] Wang Y., Wang H., Hu J., Chai J., Luan J., Li J., Xu Q. (2025). FLASH Radiotherapy: Mechanisms, Nanotherapeutic Strategy and Future Development. Nanoscale Adv..

[183] Lira M.C., Vanpouille-Box C., De Martino M. (2025). Harnessing FLASH Irradiation to Improve Immunotherapy of Medulloblastoma. Trends Cancer.

[184] Cheng C., Xu L., Jing H., Selvaraj B., Lin H., Pennock M., Chhabra A.M., Hasan S., Zhai H., Zhang Y. (2024). The Potential and Challenges of Proton FLASH in Head and Neck Cancer Reirradiation. Cancers.

[185] Ronga M.G., Gesualdi F., Bonfrate A., Patriarca A., Ferrand R., Créhange G., Buvat I., De Marzi L. (2025). Comparison of Secondary Radiation Dose between Pencil Beam Scanning and Scattered Delivery for Proton and VHEE Radiotherapy. Med. Phys..

[186] Newhauser W.D., Zhang R. (2015). The Physics of Proton Therapy. Phys. Med. Biol..

